# The shot, the message, and the messenger: COVID-19 vaccine acceptance in Latin America

**DOI:** 10.1038/s41541-021-00380-x

**Published:** 2021-09-30

**Authors:** Pablo Argote, Elena Barham, Sarah Zukerman Daly, Julian E. Gerez, John Marshall, Oscar Pocasangre

**Affiliations:** Department of Political Science, Columbia University, New York, NY USA

**Keywords:** Public health, Viral infection

## Abstract

Herd immunity by mass vaccination offers the potential to substantially limit the continuing spread of COVID-19, but high levels of vaccine hesitancy threaten this goal. In a cross-country analysis of vaccine hesitant respondents across Latin America in January 2021, we experimentally tested how five features of mass vaccination campaigns—the vaccine’s producer, efficacy, endorser, distributor, and current population uptake rate—shifted willingness to take a COVID-19 vaccine. We find that citizens preferred Western-produced vaccines, but were highly influenced by factual information about vaccine efficacy. Vaccine hesitant individuals were more responsive to vaccine messengers with medical expertise than political, religious, or media elite endorsements. Citizen trust in foreign governments, domestic leaders, and state institutions moderated the effects of the campaign features on vaccine acceptance. These findings can help inform the design of unfolding mass inoculation campaigns.

## Introduction

A rich scientific literature evaluates public health strategies aimed at containing and eradicating infectious diseases among humans. Chief among these strategies is mass vaccination^1^, which offers the potential to control the current global COVID-19 pandemic. As of June 2021, governments around the world have approved eight vaccines shown to provide protection against severe acute respiratory syndrome coronavirus 2 (SARS-CoV-2). Seventy-one more vaccines are currently in the testing pipeline awaiting approval for full use^2^. As President Joseph Biden recently announced: "COVID-19 knows no borders”^3^; “We have to end COVID-19, not just at home...but everywhere”^4^. The end of the pandemic requires global vaccination, a goal to which the U.S. and G7 countries are becoming more committed. However, achieving this goal requires not just overcoming the tenuous supply of vaccines in the developing world—resulting from global shot shortages, distribution inequities, intellectual property, and vaccine geopolitics^5^—but also addressing insufficient vaccine demand across the Global South stemming from vaccine hesitancy.

Academic research has studied the factors associated with vaccine hesitancy in the U.S. and Western Europe^6–9^. It reveals that willingness to take a vaccine in these contexts responds significantly to characteristics of the specific vaccines: high efficacy, low incidence of major adverse effects, domestic production, and endorsements from medical organizations^10–15^. However, little is yet known about the determinants of vaccine hesitancy in the developing world and specifically in virus hotspots, such as Latin America^16,17^, which ranks among the hardest hit by the COVID-19 pandemic^18^.

There are several reasons to anticipate that the determinants of vaccine acceptance could be different in Latin America than in the Global North. These are vaccine-receiving rather than vaccine-producing nations. Existing studies show that citizens prefer domestic-made over foreign-made vaccines^10,15^. However, these studies have little leverage over citizens’ COVID-19 vaccine preferences when selecting among only foreign-made vaccines (Latin America has procured vaccines from the U.S., U.K., China, Russia, and India to immunize its citizenry). Additionally, citizens in Latin America are often less informed about issues relating to public health^19,20^. Accordingly, information on vaccine safety and efficacy may prove more potent in moving vaccine preferences in that region. Compared with the U.S. and Western Europe, Latin American countries also exhibit weaker health infrastructure, requiring their governments to consider institutions beyond the health sector to distribute vaccines, and Latin American citizens possess lower trust in science such that vaccine messengers beyond medical authorities may prove more persuasive^21^. Understanding how populations perceive different aspects of vaccines in different regions of the world will ultimately be critical for designing policies to bolster vaccine acceptance and achieve quick mass inoculation globally^22^.

We undertake this task by conducting a cross-country experimental analysis of the effect of different features of mass vaccination roll out campaigns on vaccine hesitancy in six major Latin America countries where still-nascent campaigns include a variety of different vaccines and delivery methods. We use an experiment embedded in a survey of vaccine-hesitant respondents to understand how dimensions of vaccine campaigns—the vaccine producers, efficacy, endorsers, and distributors—shape the extent to which reluctant Latin Americans could be convinced to get vaccinated. While most current research measures vaccine hesitancy in a time-invariant fashion, for a speedy return to normalcy and to outpace emergent mutations that could be resistant to vaccines, it also matters *how fast* the population is willing to vaccinate. Accordingly, we measure both willingness to vaccinate and intended time to wait until inoculation. Whereas existing scholarship focuses on how public health preferences respond to partisan cues, misinformation, and hypothetical efficacy and safety facts^14,23^, we explore how these preferences also respond to alternative elite cues, to factual efficacy and safety information from clinical trials, and to varying institutions of vaccine distribution. We also compare many of these features to “unspecified” conditions, which approximate a realistic baseline where citizens are uninformed about the options available to them.

## Results

### Design

For this study, we recruited nationally representative samples of around 2000 adults each from Argentina, Brazil, Chile, Colombia, México, and Perú to participate in an online survey. These respondents, who were drawn from Netquest’s online panel, are nationally representative by age, gender, socioeconomic level, and region, according to the most recent national censuses. The survey was enumerated in late January 2021, before the roll-out of mass vaccination campaigns in Latin America. At the moment of writing, these campaigns in Latin America—and much of the Global South—remain nascent, not yet serving the general population.

The survey first elicited a respondent’s vaccine hesitancy. We measured both acceptance of a vaccine on a five-point scale ranging from strongly disagree to strongly agree and the number of months that a respondent intended to wait until vaccination. Across countries, we found that only 59% of respondents would accept a vaccine if a vaccine were available to them now and that they would wait, on average, 4.3 months to get vaccinated. We then screened out the 41% of vaccine-acceptant respondents: those who agreed or strongly agreed that they would take a vaccine and would take it within two months of becoming eligible. Descriptive statistics for this vaccine hesitant population, as well the differences between the vaccine accepting and vaccine hesitant populations in our sample, are included in Supplementary Table 2. The full survey was completed by approximately 1100 vaccine-hesitant individuals in each country—this focuses our analysis on the subset of the population whose vaccine attitudes prove most critical to understand.

We embedded a conjoint experiment^24^ in the survey to assess how different features of hypothetical—yet, at the time of enumeration, highly plausible—mass vaccine roll out scenarios affect the demand for vaccination among hesitant citizens. This experimental design randomly varied multiple features of vaccine campaigns simultaneously, allowing us to evaluate the average marginal effect of each attribute, averaging over the joint distribution of the remaining attributes. A total of 6489 respondents participated in the conjoint experiment and, because of the nature of conjoint studies, all of them were eventually exposed to the treatments. Our design replicates dimensions of vaccine conjoint experiments conducted in the U.S. and Western European contexts, but the features we study also depart and expand upon these studies in several important ways.

First, as in previous studies^10,11^, we varied the producer of the vaccine offered to respondents. However, given our focus on vaccine recipient rather than on vaccine producer countries, our comparison focused not on domestic- versus foreign-made vaccines, but rather on vaccines created by a variety of foreign countries with varying geopolitical interests and histories of intervention in the region. Latin America is the U.S.’s traditional geopolitical backyard, but experienced high levels of Russian Cold-War era intervention and recent Chinese ‘Belt and Road Initiative’ and vaccine diplomacy^25^. Accordingly, we anticipated that underlying international relations and consequent trust in foreign countries, rather than vaccine nationalism, might influence the effect of the vaccine producer on willingness to accept a vaccine.

Second, we further varied whether or not information about the efficacy of a given vaccine was provided. Unlike earlier studies in the U.S. and France, we relied not on hypothetical efficacy rates, but rather reported the Stage III trial results to evaluate the effect of learning about the actual efficacy rates of the vaccines. Respondents were randomized to receive either true trial efficacy rates of the respective vaccine or no efficacy information. The no information benchmark, which captures citizens’ prior beliefs, departs from existing studies which use as their baseline the FDA’s 50% minimum effectiveness threshold^10,11,14,15^. Our design thus enables us to disentangle the effects of vaccine efficacy from the brand of the vaccine and, given our lower public health information environment in which the population held weaker priors about vaccine efficacy, allowed us to study the merits of emphasizing efficacy rates to encourage vaccination.

Third, our design varied the endorser of the vaccines. Expanding beyond the President and medical association endorsers used in existing studies^10,11,14,15^, we also study alternative messengers: religious elites, local government authorities, and the media. These endorsers could prove more persuasive in our context of high, albeit varied, religiosity, thinner interactions with the central government, and high polarization of the news media.

Finally, we randomized information about the proportion of the population that had taken the vaccine and the institution distributing the vaccine. The former allows us to assess social effects: how the behavior of others could influence individual willingness to vaccinate^26–31^. The institution distributing the vaccine has largely been ignored by previous studies, but has been found to influence vaccine hesitancy^13,32^. We consider three actors considered for vaccine implementation in Latin America at the time we fielded our survey: the state-run public health system, the military, and civil society; private sector health and pharmaceutical facilities were not part of the distribution equation.

Table 1 summarizes all of the scenario features along each dimension; Supplementary Table 1 further lists the country-specific values of the attributes. The following script shows the translated wording which each hypothetical vaccine scenario followed:

**Table 1:** Features of mass vaccination scenario, by dimension.

Dimensions:				
	*Phase 3* *trial efficacy*			*Community* *take-up rate*
*Producer/country*		*Endorser*	*Implementer*	
Pfizer (USA)	Yes	The President	Healthcare system	1%
AstraZeneca-University of Oxford (UK)	Unspecified	Mayor of your municipality	Military	25%
Gamaleya Institute (Russia)		Medical association	Civil society	50%
Sinovax (China)		Religious leader		75%
Unspecified		Left-leaning newspaper		Unspecified
		Right-leaning newspaper

Suppose that [respondent’s country] has obtained the vaccine produced by [producer of vaccine] based in [country where the vaccine was produced]. [It has been demonstrated that the vaccine prevents [efficacy rate]% of COVID-19 infections.]

The vaccine is free of charge for everyone and [endorser] is recommending that everyone take the vaccine as soon as possible.

The vaccine is being distributed by [implementer][, and [community take up rate]% of people in your community have already been vaccinated].

Each respondent was shown five scenarios. Immediately after each scenario, respondents were asked whether, if the vaccine were available to them, they would get vaccinated, and how many months they would wait to do so (we reverse the code so positive coefficients always imply greater willingness). We also use four post-treatment measures to capture the mechanisms by which the vaccine scenario influenced willingness to vaccinate: whether the respondents believed the specific vaccine would quickly stop the spread of COVID-19; would prevent the inoculated from contracting the virus; would be unlikely to cause adverse health effects; and whether the respondents believed that the government, by mass inoculating with the vaccine, would be acting in the public interest. We leverage within-respondent variation in scenario features across rounds to increase the precision of our estimates of the average marginal effect of each feature on vaccine willingness, timing, and these four potential mechanisms.

### Analysis

**Fig. 1:**
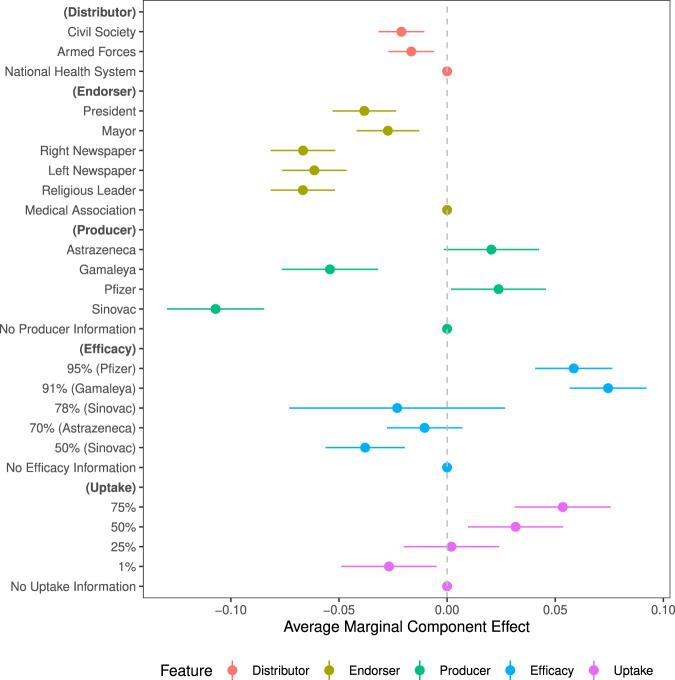
How features of a mass vaccination scenario affect willingness to take the vaccine in the scenario. *Notes*: Fig. 1 plots coefficient estimates for the full conjoint design with the outcome of respondent willingness to take the vaccine, with a binary measure of vaccine willingness. We use 95% confidence intervals, with standard errors clustered at the respondent level. The baseline categories, which are shown by the dots fixed at zero, include: national health system (distributor); national medical association (endorser); and unspecified for vaccine producer, efficacy, and uptake.

**Fig. 2:**
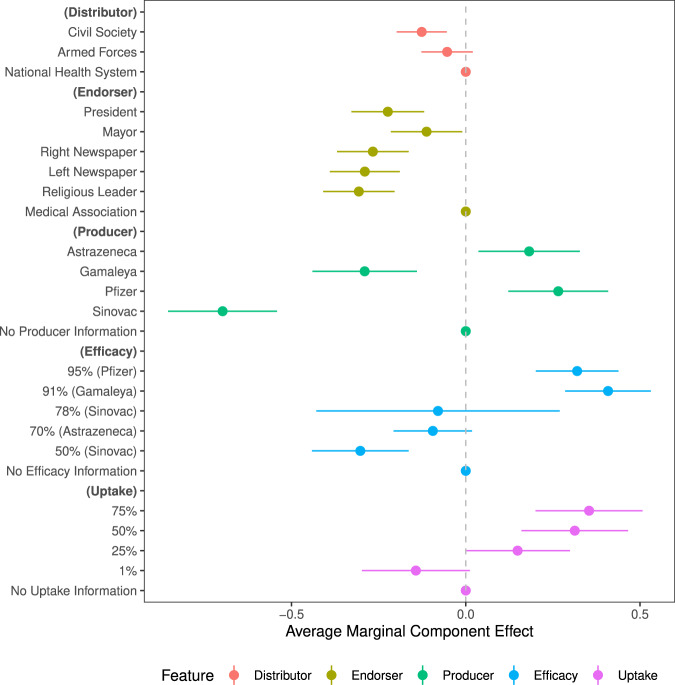
How features of a mass vaccination scenario affect how long a respondent would wait to take the the vaccine in the scenario. *Notes*: Fig. 2 plots coefficient estimates for the full conjoint design for the reported months that participants would wait prior to taking the vaccine. We use 95% confidence intervals with standard errors clustered at the respondent level. The baseline categories, which are shown by the dots fixed at zero, include: national health system (distributor); national medical association (endorser); and unspecified for vaccine producer, efficacy, and uptake. The months variable is reversed so that positive coefficients imply greater willingness.

How do different aspects of vaccines—their origin, effectiveness, uptake, endorsers, and distributors—affect acceptance rates in Latin America? Pooling the five scenarios presented to each respondent, Figs. 1 and 2 report the average marginal component effect of the campaign features on whether the respondents would get vaccinated and how long they would wait to get vaccinated. Each estimate in the figures should be read as relative to a baseline feature within each category: for vaccine producer, vaccine efficacy, and the current uptake rate, the baseline category is receiving no information on that dimension; for the endorser, the baseline category is a national medical association; and, for the distributor, the baseline category is the national public health system. We present plots for the estimated marginal means in Supplementary Fig. 1. To address concerns of waning participant focus over the course of the conjoint, we demonstrate in Supplementary Table 8 that the results are robust to restricting attention only to the first scenario encountered.

Among those reluctant to vaccinate in Latin America, hesitant respondents were sensitive to the particular vaccine on offer. Relative to a generic vaccine (that did not specify any producer), which we found 52% of respondents would be willing to take, respondents reported being 0.11 probability points—or about 20%—less likely to get vaccinated if they were offered China’s Sinovac vaccine (95% CI: −0.130 to −0.085) and 0.05 probability points less likely to get vaccinated if they were offered Russia’s Gamaleya Institute’s vaccine (95% CI: −0.077 to −0.032). As shown in Supplementary Table 6, analyses of mechanisms for why suspicion rises against these vaccines suggest that respondents are skeptical of the safety of the Sinovac and Gamaleya Institute vaccines, and, in the case of the Sinovac vaccine, would distrust their governments’ motives when inoculating with this particular vaccine—mechanisms that are quite distinct from the vaccine nationalism observed in the Global North. In contrast, citizens expressed slightly greater willingness to take a Western-produced vaccine: the UK’s AstraZeneca-Oxford vaccine increased willingness by 0.021 probability points (95% CI: −0.001 to 0.043) and the US-German Pfizer-BioNTech vaccine bolstered acceptance by 0.025 probability points (95% CI: 0.002 to 0.046). Figure 2 shows that these differences in uptake also translate into willingness to take the vaccine sooner (See Supplementary Table 8 for robustness checks).

**Fig. 3:**
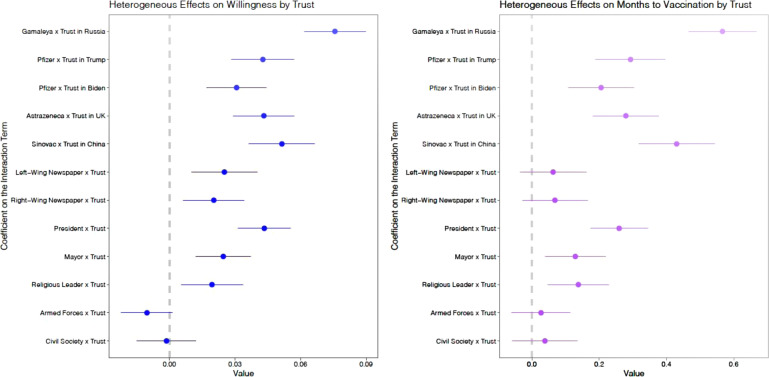
Heterogeneous effects of trust in endorsers, producers and distributors. *Notes*: Fig. 3 plots coefficient estimates for indicators of trust in the respective producer, endorser, and distributor interacted with each element of the conjoint experiment. We use 95% confidence intervals with standard errors clustered at the respondent level. Trust measures in each category range on a scale from 1 (low trust) to 4 (high trust). Coefficients should be interpreted as the change in the effect of a given endorser, distributor, or producer for a *one unit* increase in trust in the actor.

We find that vaccine hesitancy decreases significantly with the extent of trust in the government of the producer’s nation, as displayed in Fig. 3. This suggests that, while all foreign vaccines are, in theory, on equal footing in vaccine-recipient countries, in practice, underlying international relations, and consequent levels of confidence in foreign countries, play an important role in shaping vaccine preferences. Having high trust in the U.S., for example, increases willingness to take the U.S.-produced vaccine by approximately 37% of baseline willingness, relative to a generic vaccine.

Our results further show that citizens are highly sensitive to the effectiveness of vaccines^33^. Learning of the 50% efficacy rate of Sinovac further reduced vaccine acceptance, relative to receiving no information about the vaccine’s efficacy. While not statistically significant, learning of the 70% efficacy rate of the AstraZeneca-Oxford vaccine (based on its early trials) also slightly reduced vaccine willingness. These findings suggest that participants in our study may have held prior beliefs about the efficacy of a generic vaccine around 70%—such that they interpreted the treatment condition of 50% efficacy as worse than expected. The biggest gains associated with revealing efficacy relate to the Gamaleya Institute’s vaccine, for which a 91% efficacy rate increased the likelihood of taking that vaccine by 0.076 probability points (95% CI: 0.057 to 0.092). Learning of Pfizer-BioNTech’s 95% efficacy rate also increased willingness by 0.059 probability points (95% CI: 0.041 to 0.076). Despite its high efficacy, this information about the Pfizer vaccine appears to deviate less from respondents’ prior expectations than the high efficacy rate of the Russian vaccine. In these latter cases, Supplementary Table 6 shows that citizens became considerably more confident that COVID-19 would stop spreading and less concerned about individual health risks of inoculating. In sum, these results suggest that citizens discern between the vaccines on offer, and could be persuaded by factual health information to inoculate if a sufficiently appealing vaccine is available.

Our analyses also suggest that vaccine willingness in the hesitant sub-population varies with hypothetical uptake. Respondents would be 0.027 probability points less likely to get vaccinated if only 1% of their community had already been vaccinated (95% CI: −0.049 to −0.005) than if the uptake rate were unspecified. However, if 50% or 75% of the community were already vaccinated, relative to the no-information benchmark, willingness would rise by 0.031 (95% CI: 0.009 to 0.053) and 0.053 (95% CI: 0.031 to 0.075) probability points respectively. The increasing willingness of respondents to vaccinate as the percentage of the population that has been vaccinated increases is consistent with at least three mechanisms^34^: (i) social learning^26,27^, whereby individuals infer safety or efficacy of vaccines from high uptake rates^28^; (ii) social conformity, whereby individuals seeking to conform are more likely to vaccinate when the uptake rate/norm of vaccinating is higher^29–31^; and (iii) individuals becoming likely to inoculate only when they believe the campaign will be successful at reaching herd immunity^35–37^. Future research should seek to differentiate these mechanisms, which are observationally equivalent in our data. Regardless of the precise mechanism by which uptake influences inoculation preferences, the results highlight the importance of communicating high uptake rates to encourage further vaccination.

Once countries have procured specific vaccines, our design enables us to ask *who* should promote the vaccines to maximize uptake. Cues sent by different elites are known to influence public opinion and behavior in democracies^38^. Studies of the general population in the U.S. context find that endorsements from medical organizations are associated with a higher probability of choosing a vaccine than a recommendation from the President^10,14^. We find similar results among the vaccine-hesitant population in the Latin American context, where trust in science is lower^39^. Relative to a vaccine endorsement from the national medical association, willingness to get vaccinated when the President (or local Mayor) recommends the vaccine is around 0.037 (95% CI: −0.052 to −0.023) probability points lower and the wait to get vaccinated is around 0.255 (95% CI: −0.328 to −0.122) months longer. The dampening mayoral result holds in both the federal and centralized countries in our sample (with the exception of Colombia). Endorsements from actors endowed with less professional medical knowledge are least effective. Following recommendations from religious leaders and newspapers, vaccine willingness is between 0.060 and 0.068 probability points lower and the wait to get vaccinated is between 0.267 and 0.326 months longer than if the endorsement came from the country’s medical experts.

There is important heterogeneity, however, in the persuasive power of these different messengers, as shown in Supplementary Fig. 2. In particular, Evangelical respondents departed from this dominant trend. In the survey, respondents were asked their religion and, if the respondent was Catholic or Evangelical, they were then assigned to their co-religious endorser in the conjoint experiment: the Catholic archbishop (given the vertical nature of the Catholic church) or national Evangelical organization (given the far less hierarchical structure of the Evangelical church). Non-Catholics and non-Evangelicals were assigned to the Catholic archbishop of their country. In a sub-group analysis, we find that Evangelicals prove equally responsive to endorsements from their religious representatives as to recommendations from public health authorities (Supplementary Table 11). This result may explain why Brazil, the country in our sample with the largest share of Evangelicals (approximately 25%^40^), is an outlier in this regard. Education pointed in the opposite direction: relative to the general population, respondents with higher educational attainment proved significantly less responsive to endorsements from religious leaders as well as from the incumbent President, as we show in Supplementary Table 12.

Brazil and México also defied the prevailing trend of presidential endorsers proving less effective than medical ones. Existing U.S.-centric studies of COVID-19 vaccine acceptance were conducted in the shadow of the polarizing and populist Trump presidency; and yet, they found that medical authorities remained more persuasive than Trump in moving individuals to accept the COVID-19 vaccine^10,14^. The effects diverge in the Latin American countries with the most similarly populist executives who played with pandemic polarization and dismissed the severity of the coronavirus: Brazilian President Jair Bolsonaro and Mexican President Andrés Manuel López Obrador. These Presidents prove equally as effective endorsers as the national medical experts in our data. This may be because respondents find the presidential vaccine recommendation more credible since these presidents were previously more skeptical. This finding may also reflect our different sample (vaccine-hesitant versus the general population), and the higher levels of trust in and co-partisanship with the president among Mexicans and Brazilians, especially the vaccine-reluctant. Brazil and México present the highest levels of co-partisanship with the presidents in our sample, with 36% and 31% respectively, while average co-partisanship with the president across the other four countries in our study averages 11%.

We further find that trust in the messenger, and sharing the messenger’s partisan or religious identity, increases individual responsiveness to endorsements, and can improve vaccine uptake^41^. This is consistent with existing studies, which reveal the deadly role that mistrust can play in exacerbating public health crises and the life-saving role of trust^42–47^. This finding implies that, in addition to placing broadly-trusted public health professionals front and center in a national campaign, it may be advantageous to have political, religious, and media leaders publicize the vaccines directly to their voters, congregations, and readership respectively^29,48^. This approach also seems the most promising way to move the most-vaccine hesitant to vaccinate (Supplementary Table 13)^49^.

Who distributes the vaccine on the ground also proves to somewhat influence intention to vaccinate. Relative to the state’s public health system, we find negative effects on vaccine acceptance of civil society and the military distributing the vaccine, but these effects are small in magnitude. Distribution by civil society groups reduces the likelihood of taking a vaccine by 0.021 probability points (95% CI: −0.032 to −0.011) and distribution by the military reduces this likelihood by 0.017 probability points (95% CI: −0.027 to −0.006). Given weaker state capacity in developing country contexts, this finding indicates that utilizing all of these institutional hands on deck may be the most promising way to mass mobilize to inoculate in these settings.

Again, interesting heterogeneity emerges, specifically with respect to the military as a vaccine-distributor. The data suggests a significant negative effect of the armed forces as a vaccine distributor in Colombia and Chile. We interpret this result as reflecting the fact that, whereas all countries in our sample have a history of dictatorship and conflict, in these two countries in particular, the armed forces have been implicated in repression against peaceful protests in the past two years, sparking a backlash against this institution. Similar protests and military crackdowns have roiled other parts of the world, suggesting that armed forces’ involvement in vaccine distribution could play a similarly dampening effect on vaccine acceptance in these contexts.

## Discussion

To end the global health crisis, it is necessary to ensure not only that everyone in the world has access to COVID-19 vaccines, but also that populations everywhere are willing to take them, and to do so quickly enough to mitigate the risk of emergent vaccine-resistant mutations^50^. However, research on vaccine acceptance thus far has predominantly concentrated on the U.S. and Western Europe. Following a systematic search of the peer-reviewed English survey literature on vaccine acceptance indexed in PubMed, scholars have concluded that studies of COVID-19 vaccine hesitancy are urgently needed in the developing world, including in South and Central America^16^. We contribute one such study.

Given that vaccine production is concentrated in the Global North, governments in the Global South do not have their pick of vaccines. We find that vaccine hesitancy in Latin America is not uniform; rather, it proves highly responsive to which vaccine is on offer. We observe strong evidence that citizens privilege Western-produced vaccines. This is worrisome as many countries in this region are procuring a diverse portfolio of vaccines, including non-Western ones, to secure as many vaccines as quickly as possible. Governments of vaccine-recipient countries should be wary of viewing all vaccines as a panacea to the continued spread and devastation of COVID-19; instead, reluctance to take certain vaccines may hamper campaigns armed with such shots.

However, our evidence provides an actionable antidote to counter suspicions of non-Western vaccines: efficacy information. Albeit a double-edged sword, if the vaccines are effective, simple and clear facts highlighting these levels of efficacy as well as others’ uptake of the vaccine—particularly if high—could significantly increase citizen willingness to immunize.

In this sense, our results depart from public health studies that find little increase in vaccine acceptance due to dispelling myths and misinformation^51,52^. Our data suggest, in an environment where citizens possess imprecise prior beliefs about issues of public health, a powerful ability of factual information about vaccine effectiveness to convince the hesitant to inoculate^7,53^, although misinformation may still matter^6^. Hesitant citizens’ responsiveness to efficacy information suggests that, to ensure that uptake in the Global South crosses the threshold needed to realize the goal of global herd immunity, industrialized countries will need to provide not only a large quantity of vaccines to developing countries through initiatives such as the World Health Organization’s COVAX, but also high-quality ones, and that developing countries, in turn, should seek to procure such high-efficacy shots^54^. While our study focused on the overall proven efficacy rates from Stage III trials, future research should seek to disaggregate these numbers to understand how hesitancy responds to vaccines’ effectiveness against minor versus severe illness, hospitalization, and death, and against virus mutations. This may be especially important when considering the vaccines that are most effective against severe COVID-19.

Our results align with research underscoring the important role of national medical elite cues to promote vaccines in the Global North^10,14,29,55^. This may seem surprising given the greater skepticism of science and elevated influence of alternative sources of authority in Latin America. At the same time, we find that, in a world of echo chambers, citizens appear most likely to listen to cues from in-group members^56,57^. For ethical reasons, we considered only pro-vaccine elite cues. Caught in the real-world cacophony and cross-fire between vaccine endorsements and criticisms, citizens may respond differently to the messengers than in our controlled environment^58^.

Our study focused on the vaccine hesitant. However, it is possible that the campaign messaging could backfire among the vaccine acceptant. Based on Supplementary Figs. 3 through 8, we posit that this is unlikely; among the general population, average trust levels—particularly in China and in their national president—are higher than among the hesitant population, and can therefore mitigate the negative effects of certain shots, messages, and messengers on vaccine acceptance.

Overall, our model of citizen vaccine demand in Latin America has significant implications for the design of unfolding mass campaigns aimed at inducing swift and broad public vaccine uptake to substantially reduce morbidity and mortality from COVID-19 in the region, and to end the pandemic globally.

## Materials and methods

This study was approved by Columbia University’s Institutional Review Board (protocol number IRB-AAAT5273). It complies with all relevant ethical regulations for work with human participants. Written informed consent was obtained. The design and core estimation strategies were registered in a pre-analysis plan deposited in the Social Science Registry (socialscienceregistry.org/trials/7080). All statistical analyses were implemented in R.

### Recruitment

For our single-wave study, we recruited around 2000 adults from large online panels in each of Argentina, Brazil, Chile, Colombia, México, and Perú. Respondents in each country were recruited via Netquest’s online panels between January 11 and February 2, 2021. Netquest maintains large panels of survey respondents in most Latin American countries, including at least 125000 panelists in all six countries in this study. Panelists are regularly invited to take surveys, although this is not their primary vocation. Netquest’s dynamic enrollment updated invitations to ensure that the sample frame was nationally representative in terms of sex, age category, socioeconomic status, and region. Upon clicking a link to participate, respondents reached a Qualtrics (January 2021 version) landing page, where information about the academic study was provided and consent to participate in the study was obtained. With the exception of lower socioeconomic status respondents in México and Perú, the marginal distribution of respondents that started the survey (i.e., reached our screening juncture) closely approximated the census distribution for most country-variables. Given the online nature of the survey, respondents may not be representative on other dimensions, such as urban/rural location or access to fast internet.

### Screening

In addition to screening out respondents who were already willing to take a vaccine within 2 months of it becoming available, we also screened out respondents aged below 18 (*n* = 9) and those who failed our attention check eleven questions into the main survey (by failing to correctly identify the capital city of their country; *n* = 11). Given the limited screening of respondents, our sample of hesitant respondents is also likely to be broadly nationally representative of the vaccine hesitant subgroup. The median completed survey lasted 26 min; those that completed the survey were compensated with approximately 3 US dollars. Respondents who took less than 10 min to complete the survey (*n* = 47) were excluded from the analysis.

### Experimental design

Within the conjoint experiment, each respondent was shown five scenarios, with feature assignments blocked by prior vaccine willingness and age group within each country. The unspecified category could be observed only in the first round, to prevent respondents from receiving an unspecified feature after being shown specific information on that dimension in a prior round. As described in the main text and Table 1, the experimental design varied five attributes of the distribution scenario: (1) the vaccine distributor; (2) the vaccine (including country of origin); (3) whether information was given about the efficacy rate; (4) an endorser of the vaccine; and (5) levels of population uptake. Among these, the distributors and endorsers were country-specific organizations. Supplementary Table 1 presents the country-specific values of these different attributes.

Following each scenario, respondents were asked a series of questions that make up our outcome variables. These questions were: “If this vaccine were available to you, would you get it?” and “If the vaccine were available to you, how many months would you wait to get it?” Finally, in an effort to understand the underlying mechanisms, respondents were then asked “If this vaccine were available to you, to what extent do you agree with the following statements?” The list of statements included: The spread of COVID-19 will end quickly; it would be very unlikely that I would get COVID-19 if I get this vaccine; it would be very unlikely that I have a side-effect if I receive this vaccine; the government’s vaccination program is meant to help its citizens.

### Estimation

We estimate the average marginal component effect of each feature, relative to a baseline category within each dimension, by estimating the following pre-specified OLS regressions:

1 $$\begin{array}{ll}{Y}_{{{{\rm{irc}}}}}={\alpha }_{{{{\rm{brc}}}}}+{\beta }_{r}{Y}_{{{{\rm{ic}}}}}^{{{{\rm{pre}}}}}+{\gamma }_{i}+\mathop{\sum }\limits_{k=1}^{4}{\tau }_{1}^{k}{{{\rm{Producer}}}}\,{{{{\rm{k}}}}}_{{{{\rm{irc}}}}}+\mathop{\sum }\limits_{k=1}^{4}{\tau }_{3}^{k}{{{\rm{Producer}}}}\,{{{\rm{k}}}}\,{{{\rm{and}}}}\,{{{{\rm{efficacy}}}}}_{{{{\rm{irc}}}}}\\ \qquad\;+\mathop{\sum }\limits_{k=1}^{5}{\tau }_{3}^{k}{{{\rm{Endorser}}}}\,{{{{\rm{k}}}}}_{{{{\rm{irc}}}}}+\mathop{\sum }\limits_{k=1}^{2}{\tau }_{4}^{k}{{{\rm{Distributor}}}}\,{{{{\rm{k}}}}}_{{{{\rm{irc}}}}}+\mathop{\sum }\limits_{k=1}^{4}{\tau }_{5}^{k}{{{\rm{Takeup}}}}\,{{{\rm{rate}}}}\,{{{{\rm{k}}}}}_{{{{\rm{irc}}}}}+{\varepsilon }_{{{{\rm{irc}}}}},\end{array}$$

where *Y*_irc_ is an outcome in conjoint scenario round *r* for respondent *i* from country *c*, *α*_brc_ are block × round × country fixed effects, ${Y}_{{{{\rm{ic}}}}}^{{{{\rm{pre}}}}}$ measures pre-treatment immediacy of vaccine uptake (with an effect allowed to vary by round, wherever relevant, a lagged outcome), and *γ*_*i*_ are respondent fixed effects. Producer, take up rate, and efficacy all have a pure control condition, in which no producer, efficacy, or take-up rate is specified. Distributor and endorser have no non-specific control attribute, so we estimate the effects of different distributors and endorsers relative to the medical sector as a baseline in both cases: the national health system as a distributor, and the national medical association as the endorser. We include design-based inverse probability of treatment weights to account for differences in the probabilities of attribute assignment across rounds, which emerge as respondents can only be assigned an unspecified attribute in the first conjoint scenario, leading to a diminishing number of pure control scenarios as conjoint rounds progress. We cluster our standard errors at the individual level, to account for individual autocorrelation across response rounds. All statistical inferences are derived from two-tailed t tests and 95% confidence intervals based on the regressions previously described.

Our estimates can be interpreted causally under the following assumptions: (i) the assignment of features is ignorable and independent across features; and (ii) the response of a respondent exhibits stability across scenarios and are not affected by prior scenarios^24^. Our independent randomization of attributes (within rounds) ensures that the assignment of each attribute is, in expectation, independent of potential outcomes and the assignment of other attributes. Suggesting that this assumption indeed holds, Supplementary Table 4 shows that the attributes within each dimension are generally uncorrelated with predetermined covariates that could influence the response to each post-treatment question. The first assumption further requires that respondent attrition is orthogonal to the attributes presented in the scenario after which attrition occurs, which we provide empirical support for in Supplementary Table 5. The stable unit treatment value assumption (SUTVA) is supported by the results in Supplementary Table 8; the table shows that the estimates from the first scenario that a respondent encountered are similar, if less precisely estimated, to the results that pool across scenarios.

#### Estimating and interpreting heterogeneous effects of respondent traits on uptake

In this paper, we present heterogeneous treatment effects by trust as well as by other characteristics of respondents, including co-partisanship, religious denomination, education, and level of pre-treatment hesitancy. Here we present a generic equation for estimating heterogeneous effects, in which we use the variable *X* *k*_*i*_ to capture a generic moderating variable. In-line with best practice^59^, we interpret all heterogeneous effects as indicative of differences in the magnitude of the treatment effect across the subgroups described in the conditioning variable, and not indicative of descriptive differences in preferences of one sub-group relative to another.

2 $$\begin{array}{lll}{Y}_{{{{\rm{irc}}}}}&=&{\alpha }_{{{{\rm{brc}}}}}+{\beta }_{r}{Y}_{{{{\rm{ic}}}}}^{{{{\rm{pre}}}}}+{\gamma }_{i}+\mathop{\sum }\limits_{k=1}^{4}{\tau }_{1}^{k}{{{\rm{Producer}}}}\,{{{{\rm{k}}}}}_{{{{\rm{irc}}}}}\\ &&+\,\mathop{\sum }\limits_{k=1}^{4}{\tau }_{3}^{k}{{{\rm{Producer}}}}\,{{{\rm{k}}}}\,{{{\rm{and}}}}\,{{{{\rm{efficacy}}}}}_{{{{\rm{irc}}}}}+\mathop{\sum }\limits_{k=1}^{5}{\tau }_{3}^{k}{{{\rm{Endorser}}}}\,{{{{\rm{k}}}}}_{{{{\rm{irc}}}}}\\ &&+\mathop{\sum }\limits_{k=1}^{2}{\tau }_{4}^{k}{{{\rm{Distributor}}}}\,{{{{\rm{k}}}}}_{{{{\rm{irc}}}}}+\mathop{\sum }\limits_{k=1}^{4}{\tau }_{5}^{k}{{{\rm{Takeup}}}}\,{{{\rm{rate}}}}\,{{{{\rm{k}}}}}_{{{{\rm{irc}}}}}\\ &&+\mathop{\sum }\limits_{k=1}^{4}{\tau }_{1}^{k}{{{\rm{Producer}}}}\,{{{{\rm{k}}}}}_{{{{\rm{irc}}}}}\,\times X\,{k}_{{{{\rm{i}}}}}\\ &&+\mathop{\sum }\limits_{k=1}^{4}{\tau }_{3}^{k}{{{\rm{Producer}}}}\,{{{\rm{k}}}}\,{{{\rm{and}}}}\,{{{{\rm{efficacy}}}}}_{{{{\rm{irc}}}}}\,\times X\,{k}_{{{{\rm{i}}}}}\\ &&+\mathop{\sum }\limits_{k=1}^{5}{\tau }_{3}^{k}{{{\rm{Endorser}}}}\,{{{{\rm{k}}}}}_{{{{\rm{irc}}}}}\times X\,{k}_{{{{\rm{i}}}}}\\ &&+\mathop{\sum }\limits_{k=1}^{2}{\tau }_{4}^{k}{{{\rm{Distributor}}}}\,{{{{\rm{k}}}}}_{{{{\rm{irc}}}}}\,\times X\,{k}_{{{{\rm{i}}}}}\\ &&+\mathop{\sum }\limits_{k=1}^{4}{\tau }_{5}^{k}{{{\rm{Takeup}}}}\,{{{\rm{rate}}}}\,{{{{\rm{k}}}}}_{{{{\rm{irc}}}}}\,\times X\,{k}_{{{{\rm{i}}}}}+{\varepsilon }_{{{{\rm{irc}}}}}.\end{array}$$

As in equation (1), *Y*_irc_ is the outcome in conjoint scenario round *r* for respondent *i* from country *c*, and *α*_*bcr*_ are block × round × country fixed effects, ${Y}_{{{{\rm{ic}}}}}^{{{{\rm{pre}}}}}$ measures the pre-treatment immediacy of vaccine uptake (with an effect allowed to vary by round, wherever relevant, a lagged outcome), and *γ*_*i*_ are respondent fixed effects. We again include inverse probability of treatment weights to account for the differences in the probabilities of treatment across rounds, as in the main estimation, and we cluster our standard errors at the individual level, to account for individual autocorrelation across response rounds.

For the analysis in Supplementary Table 9 and Fig. 3, *X k*_*i*_ takes on the value of a respondents’ trust in the corresponding conjoint attribute (Producer, Endorser, and Distributor). There are no interactions for the *Producer k and efficacy* or *Takeup rate* elements of the conjoint. All trust measures are drawn directly from pre-treatment questions which quantify trust on a four point scale from very low to very high trust.

### Reporting summary

Further information on research design is available in the Nature Research Reporting Summary linked to this article.

## Supplementary information

Supplementary Information

Reporting Summary

## Data Availability

All data needed to evaluate the conclusions in the paper are present in the paper and/or the Supplementary Materials publicly available online (https://github.com/sarahzdaly/Vaccine-Acceptance-in-Latin-America). The Spanish language survey instrument is provided there; the Portuguese version is available upon request from the authors.

## Code availability

The code developed for this study is available at https://github.com/sarahzdaly/Vaccine-Acceptance-in-Latin-America.
