# Supplementary material for: The shot, the message, and the messenger: COVID-19 vaccine acceptance in Latin America
Source: NPJ Vaccines. 2021 Sep 30;6:118. doi: 10.1038/s41541-021-00380-x (PMC8484594; doi:10.1038/s41541-021-00380-x)
Supplement: Supplementary file 1 — Supplementary Information [file 41541_2021_380_MOESM1_ESM.pdf]

Supplementary Materials for

The Shot, The Message and the Messenger: COVID-19 Vaccine Acceptance in Latin America

Pablo Argote, Elena Barham, Sarah Zukerman Daly*, Julian E. Gerez, John Marshall, and Oscar Pocasangre

*Corresponding Author, sd2623@columbia.edu

Supplementary Materials

Supplementary Table 1: Country-Specific Attributes of Mass-Vaccination Campaigns

**Category**	*Argentina*	*Brazil*	*Chile*
National Medical Association	Asociación Médica Argentina	Conselho Federal de Medicina	Colegio Médico de Chile
President	Alberto Fernández	Jair Bolsonaro	Sebastián Piñera
Mayor	Intendente	Prefeito	Alcalde
Catholic Authority	Cardenal Mario Aurelio Pol	Cardeal Sérgio da Rocha	Arzobispo Celestino Aós
Evangelical Authority	Alianza Cristiana de Iglesias Evangélicas de la República de Argentina	Aliança Cristã Evangélica Brasileira	Mesa Ampliada - Unión Nacional Evangélica
Left-Leaning Newspaper	El Clarín	Folha de Sao Paolo	La Tercera
Right-Leaning Newspaper	La Nación	O Globo	El Mercurio
	Colombia	*Mexico*	*Peru*
National Medical Association	Federación Médica Colombaina	Academia Nacional de Medicina	Colegio Médico del Perú
President	Iván Duque	Andrés Manuel López Obrador	Francisco Sagasti
Mayor	Alcalde	Alcalde	Alcalde
Catholic Authority	Arzobispo Luis José Rueda	Arzobispo Carlos Aguiar Retes	Arzobispo Carlos Castillo Mattasoglio
Evangelical Authority	Confederación Evangélica de Colombia (CEDECOL)	Confraternidad Evangélica de México (CONEMEX)	Unión Nacional de Iglesias Cristianas Evangélicas del Perú (UNICEP)
Left-Leaning Newspaper	El Espectador	La Jornada	La República
Right-Leaning Newspaper	El Tiempo	Reforma	El Comercio

Per-Country Hesitancy and Demographic Information of the Hesitant and Non-Hesitant

Countries in our sample varied in their per-country hesitancy level. The most hesitant country in our sample was Chile with 50% willingness at the time of our survey. Argentina (56%), Colombia (58%), México (66%), and Perú (51%) displayed intermediate levels of hesitancy. Brazil was the most vaccine acceptant, with 68% willingness at the time of our survey. Here we display descriptive data on traits of our vaccine hesitant, in comparison with a smaller set of data collected about traits of non-hesitant respondents.

Supplementary Table 2: Demographic Information for Hesitant and Non-Hesitant Populations

	Demographic Characteristics					
	**Hesitant**		**Accepting**		**Survey Total**	
	No.	*Percent*	*No.*	*Percent*	*No.*	*Percent*
**Age (Years)**						
18-29	2524	0.33	1315	0.24	3839	0.30
30-44	2305	0.3	1473	0.27	3778	0. 29
45-59	1777	0.23	1382	0.25	3159	0.25
60+	1130	0.15	969	0.18	2099	0.16
**Sex**						
Male	3558	0.46	3084	0.57	6642	0.49
Female	4193	0.54	2759	0.51	6952	0.51
**Educational Attainment**						
None	157	0.02	82	0.02	239	0.02
Primary	529	0.07	305	0.06	834	0.06
Secondary	3567	0.45	2127	0.39	5694	0.44
University	2150	0.28	1755	0.32	3905	0.30
Other Higher Degree	1433	0.19	768	0.14	2201	0.17
**SES**						
Low	2853	0.37	1697	0.31	4550	0.35
Middle	4224	0.55	3078	0.57	7302	0.55
High	655	0.08	656	0.12	1311	0.10

Supplementary Table 3: Descriptive Information of the Hesitant

	Demographic Characteristics	
	**Hesitant**	
	No.	*Percent*
**Religion**		
Catholic	4289	0.55
Evangelical	965	0.12
None	1189	0.15
Other	1293	0.17
**Ideology**		
Left	1130	0.15
Center	5092	0.66
Right	1162	0.15
**Vote Intention**		
Incumbent	1328	0.17
Opposition	2188	0.28
Wouldn't vote	2421	0.31
Doesn't know	1422	0.18
**General Vaccine Acceptance**		
Has rejected vaccines for a child	1262	0.16
**Thinks Covid is serious**		
Yes	6569	0.85
No	1167	0.15
**Covid Diagnosis**		
Yes	1002	0.13
No	6734	0.87
**Risk Factors**		
Has one or more comorbidities	2289	0.3
No comorbidities	5447	0.7

Balance Across Conjoint Randomization

We conduct balance tests to ensure that the randomization in the conjoint experiment yielded a balance across treatment conditions. The largely insignificant differences across treatment conditions, as shown in Table 4, indicate that the groups exposed to different treatment conditions were not systematically different.

Supplementary Table 4: Covariate Balance in the First Round of the Conjoint Experiment

	Dependent variable:				
	Age Bin	Gender	Education	Pre-Treatment Hesitancy	Pre-Treatment Months
	(1)	(2)	(3)	(4)	(5)
Distributor: Civil Society	0.042	-0.030^{*}	0.002	0.078^{*}	−0.033
	(0.054)	(0.017)	(0.033)	(0.043)	(0.032)
Distributor: Armed Forces	−0.037	0.018	-0.070^{**}	−0.049	−0.047
	(0.054)	(0.017)	(0.033)	(0.043)	(0.032)
Endorser: Religious Leader	0.021	−0.023	0.093^{**}	−0.017	−0.023
	(0.077)	(0.024)	(0.047)	(0.061)	(0.046)
Endorser: Mayor	0.058	−0.018	−0.013	−0.015	-0.111^{**}
	(0.075)	(0.023)	(0.046)	(0.060)	(0.045)
Endorser: President	−0.010	0.009	0.016	−0.014	−0.032
	(0.076)	(0.023)	(0.046)	(0.060)	(0.045)
Endorser: Right Newspaper	0.101	0.050^{**}	0.001	0.038	−0.028
	(0.075)	(0.023)	(0.046)	(0.060)	(0.045)
Endorser: Left Newspaper	−0.124	0.037	0.016	0.025	0.024
	(0.076)	(0.024)	(0.046)	(0.060)	(0.045)
Producer: Sinovac	0.001	−0.047	0.067	−0.068	0.016
	(0.108)	(0.033)	(0.065)	(0.085)	(0.064)
Producer: Astrazeneca	0.023	−0.031	0.039	−0.032	−0.074
	(0.110)	(0.034)	(0.067)	(0.087)	(0.065)
Producer: Pfizer	0.076	−0.008	0.149^{**}	−0.030	−0.061
	(0.108)	(0.033)	(0.066)	(0.086)	(0.064)
Producer: Gamaleya	0.051	−0.020	0.076	−0.042	0.046
	(0.108)	(0.033)	(0.066)	(0.086)	(0.064)
1% Uptake	−0.026	-0.052^{**}	0.076^{*}	−0.029	−0.043
	(0.071)	(0.022)	(0.043)	(0.056)	(0.042)
25% Uptake	−0.058	−0.025	0.028	−0.039	−0.056
	(0.069)	(0.021)	(0.042)	(0.055)	(0.041)
50% Uptake	0.027	−0.009	−0.026	−0.038	-0.096^{**}
	(0.070)	(0.022)	(0.042)	(0.055)	(0.042)
75% Uptake	−0.030	-0.064^{***}	0.053	0.006	-0.076^{*}
	(0.070)	(0.022)	(0.043)	(0.056)	(0.042)
Efficacy Concern	−0.071	−0.029	0.015	0.009	−0.068
	(0.105)	(0.032)	(0.064)	(0.083)	(0.063)
50% Efficacy	−0.063	−0.013	0.002	0.014	0.087
	(0.106)	(0.033)	(0.064)	(0.084)	(0.063)
70% Efficacy	0.229	0.172^{***}	−0.130	−0.175	−0.006
	(0.203)	(0.063)	(0.123)	(0.161)	(0.121)
78% Efficacy	−0.024	−0.039	-0.137^{**}	−0.043	−0.023
	(0.105)	(0.033)	(0.064)	(0.084)	(0.063)
91% Efficacy	−0.063	−0.024	-0.157^{**}	0.074	0.025
	(0.104)	(0.032)	(0.063)	(0.083)	(0.062)
Fixed Effects	Yes	Yes	Yes	Yes	Yes
Outcome Range	1-6	0-1	1-5	1-5	-0.96-1.732
Control Mean	2.932	0.525	3.627	3.034	0.633
Control SD	1.639	0.504	1.081	1.402	0.971
Observations	5,317	5,317	5,317	5,317	5,317
R^2	0.018	0.015	0.083	0.032	0.024
Note:					*p<0.1; **p<0.05; ***p<0.01

In some experimental designs, non-random attrition of study participants can generate a threat to experimental validity. In this study, we assign treatment independently across rounds. Differential attrition across the course of the conjoint rounds thus does not represent a challenge to the validity of our experiment. In Supplementary Table 5, we test whether the outcome in a given round k of the conjoint is missing as a function of the attributes in round k. We find that non-response

is slightly more likely when respondents are exposed to the endorsement of a mayor relative to a health professional, but in no other condition is attrition significantly different from in our baseline categories.

Supplementary Table 5: Testing for Differential Attrition Across Conjoint Conditions

	Dependent variable:	
	Attrition - Willing	Attrition - Months
	(1)	(2)
Distributor: Civil Society	0.0002	0.0002
	(0.001)	(0.001)
Distributor: Armed Forces	0.0003	0.0003
	(0.001)	(0.001)
Endorser: Religious Leader	0.0003	0.0003
	(0.002)	(0.002)
Endorser: Mayor	0.004**	0.004**
	(0.002)	(0.002)
Endorser: President	−0.001	−0.001
	(0.002)	(0.002)
Endorser: Right Newspaper	0.003	0.003
	(0.002)	(0.002)
Endorser: Left Newspaper	0.004	0.004
	(0.002)	(0.002)
Producer: Sinovac	0.005	0.005
	(0.003)	(0.003)
Producer: Astrazeneca	0.003	0.003
	(0.003)	(0.003)
Producer: Pfizer	0.001	0.001
	(0.003)	(0.003)
Producer: Gamaleya	0.003	0.003
	(0.003)	(0.003)
1% Uptake	0.0004	0.0004
	(0.003)	(0.003)
25% Uptake	−0.0003	−0.0003
	(0.003)	(0.003)
50% Uptake	0.0001	0.0001
	(0.003)	(0.003)
75% Uptake	0.0003	0.0003
	(0.003)	(0.003)
Efficacy Concern	−0.001	−0.001
	(0.002)	(0.002)
50% Efficacy	−0.0003	−0.0003
	(0.002)	(0.002)
70% Efficacy	−0.005	−0.005
	(0.004)	(0.004)
78% Efficacy	0.002	0.002
	(0.002)	(0.002)
91% Efficacy	0.001	0.001
	(0.003)	(0.003)
Fixed Effects	Yes	Yes
Outcome Range	0-1	0-1
Control Mean	0.02	0.02
Control SD	0.141	0.141
Observations	32,017	32,017
R^2	0.565	0.565
Note:		*p<0.1; **p<0.05; ***p<0.01

Results of Basic Conjoint Experiment

In this section we present the estimated marginal means plots for our conjoint treatment condition, as suggested by [31], as well as the regression output associated with our main conjoint tables in the body of the paper. Estimated marginal means offer the benefit of an estimate that is not defined relative to a baseline category. For this reason, however, the estimated marginal means do not test the causal effect of a given conjoint attribute relative to a baseline. To yield a more complete understanding of descriptive differences in our results, we present the estimated marginal means here as a complement to the AMCEs presented in the main text. Supplementary Figure 1 presents the estimated marginal means for our conjoint treatment condition.

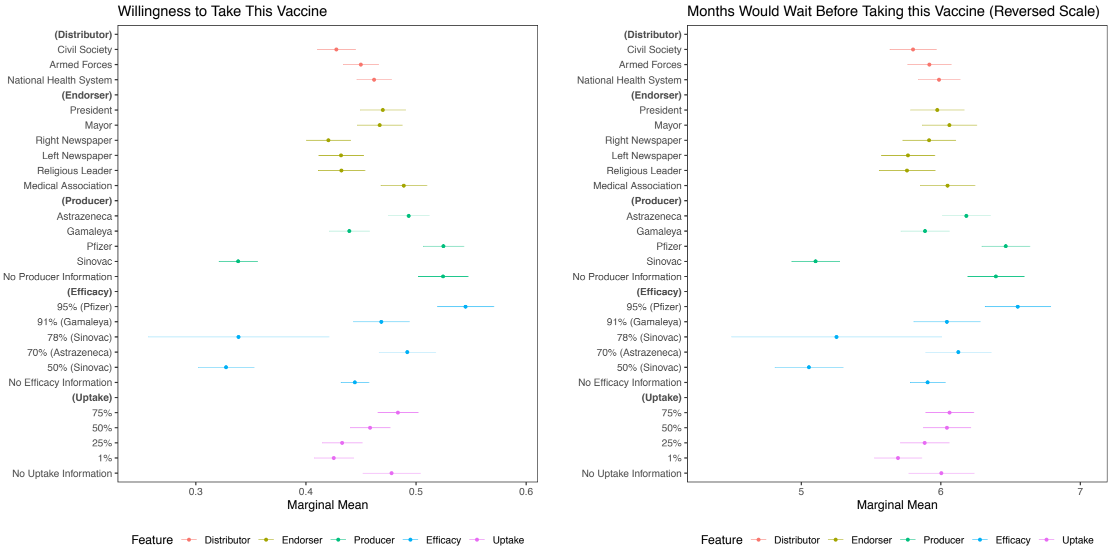

Supplementary Figure 1: Estimated Marginal Means

The results presented in Supplementary Table 6 follow Equation 1, the estimator underlying our figures 1 and 2 in the main text, but with distinct Y_{irc} in each column. Columns 1 and 2 present our core outcome measures, willingness to take the vaccine and months to vaccination (reversed for ease of interpretation) respectively. Columns (3)-(6) are post-conjoint questions about mechanisms which may shift hesitancy. These columns include: (3) The propagation of COVID-19 will stop quickly; (4) It's unlikely that I will get COVID-19 if I get this vaccine; (5) It's unlikely that I will suffer harm from getting this vaccine; (6) The government's purpose for this vaccination campaign is to help people. Answers to these mechanism questions fall on a five point scale, from strongly disagree to strongly agree.

Supplementary Table 6: Results of the Conjoint Experiment (All Rounds)

	Dependent variable:					
	Willing	Months (Rev)	Stop Propagation	Not Get COVID	Wouldn't Harm	Gov Help
	(1)	(2)	(3)	(4)	(5)	(6)
Distributor: Civil Society	−0.021***	−0.127***	−0.011	−0.020*	−0.024**	−0.018
	(0.005)	(0.037)	(0.012)	(0.012)	(0.011)	(0.011)
Distributor: Armed Forces	−0.017***	−0.053	−0.011	−0.006	−0.015	0.003
	(0.005)	(0.038)	(0.012)	(0.012)	(0.011)	(0.011)
Endorser: Religious Leader	−0.068***	−0.326***	−0.092***	−0.086***	−0.074***	−0.076***
	(0.008)	(0.053)	(0.017)	(0.018)	(0.017)	(0.016)
Endorser: Mayor	−0.026***	−0.114**	−0.055***	−0.006	−0.018	−0.010
	(0.007)	(0.052)	(0.016)	(0.017)	(0.016)	(0.015)
Endorser: President	−0.037***	−0.225***	−0.071***	−0.038**	−0.045***	−0.031**
	(0.007)	(0.052)	(0.017)	(0.017)	(0.016)	(0.014)
Endorser: Right Newspaper	−0.065***	−0.267***	−0.089***	−0.063***	−0.056***	−0.060***
	(0.008)	(0.052)	(0.016)	(0.017)	(0.016)	(0.015)
Endorser: Left Newspaper	−0.060***	−0.291***	−0.096***	−0.049***	−0.049***	−0.050***
	(0.007)	(0.051)	(0.016)	(0.017)	(0.016)	(0.015)
Producer: Sinovac	−0.107***	−0.698***	−0.151***	−0.114***	−0.130***	−0.129***
	(0.011)	(0.080)	(0.026)	(0.026)	(0.026)	(0.024)
Producer: Astrazeneca	0.021*	0.182**	0.043	0.021	0.016	0.060**
	(0.011)	(0.074)	(0.026)	(0.026)	(0.026)	(0.023)
Producer: Pfizer	0.024**	0.265***	0.031	0.060**	0.036	0.009
	(0.011)	(0.073)	(0.026)	(0.026)	(0.026)	(0.024)
Producer: Gamaleya	−0.054***	−0.291***	−0.027	−0.034	−0.049*	−0.035
	(0.011)	(0.077)	(0.026)	(0.026)	(0.026)	(0.024)
1% Uptake	−0.027**	−0.145*	−0.026	−0.022	−0.015	−0.004
	(0.011)	(0.079)	(0.026)	(0.027)	(0.026)	(0.023)
25% Uptake	0.002	0.147*	0.036	0.021	0.023	0.038*
	(0.011)	(0.077)	(0.026)	(0.027)	(0.025)	(0.022)
50% Uptake	0.031***	0.311***	0.084***	0.045*	0.046*	0.060**
	(0.011)	(0.078)	(0.027)	(0.028)	(0.025)	(0.023)
75% Uptake	0.053***	0.352***	0.090***	0.069**	0.053**	0.071***
	(0.011)	(0.078)	(0.026)	(0.027)	(0.026)	(0.023)
50% Efficacy	−0.038***	−0.301***	−0.133***	−0.128***	−0.040*	−0.051***
	(0.009)	(0.071)	(0.022)	(0.022)	(0.021)	(0.019)
70% Efficacy	−0.010	−0.094	−0.007	−0.013	0.018	−0.023
	(0.009)	(0.058)	(0.020)	(0.019)	(0.019)	(0.017)
78% Efficacy	−0.023	−0.080	−0.057	−0.046	−0.006	0.057
	(0.025)	(0.178)	(0.057)	(0.046)	(0.048)	(0.048)
91% Efficacy	0.075***	0.409***	0.090***	0.112***	0.094***	0.085***
	(0.009)	(0.063)	(0.019)	(0.020)	(0.018)	(0.017)
95% Efficacy	0.058***	0.319***	0.125***	0.084***	0.051***	0.083***
	(0.009)	(0.061)	(0.020)	(0.020)	(0.019)	(0.018)
Fixed Effects	Yes	Yes	Yes	Yes	Yes	Yes
Outcome Range	0-1	0-12	1-5	1-5	1-5	1-5
Control Mean	0.507	5.521	2.849	2.959	2.945	3.315
Control SD	0.503	4.463	1.186	1.16	1.246	1.212
Observations	31,574	31,574	31,574	31,574	31,574	31,574
R^2	0.703	0.833	0.718	0.680	0.678	0.768

Note: *p<0.1; **p<0.05; ***p<0.01

We also present the confidence intervals around point estimates of conjoint component effects, as referenced in the body of the text.

Supplementary Table 7: Conjoint Results (Confidence Intervals)

	Dependent variable:					
	Willing	Months (Rev)	Stop Propagation	Not Get COVID	Wouldn't Harm	Gov Help
	(1)	(2)	(3)	(4)	(5)	(6)
Distributor: Civil Society	−0.021***	−0.127***	−0.011	−0.020*	−0.024**	−0.018
	(−0.032, −0.011)	(−0.198, −0.055)	(−0.034, 0.013)	(−0.045, 0.004)	(−0.047, −0.002)	(−0.038, 0.003)
Distributor: Armed Forces	−0.017***	−0.053	−0.011	−0.006	−0.015	0.003
	(−0.027, −0.006)	(−0.127, 0.021)	(−0.035, 0.012)	(−0.029, 0.018)	(−0.037, 0.007)	(−0.018, 0.024)
Endorser: Religious Leader	−0.068***	−0.326***	−0.092***	−0.086***	−0.074***	−0.076***
	(−0.083, −0.053)	(−0.430, −0.222)	(−0.126, −0.059)	(−0.122, −0.051)	(−0.108, −0.040)	(−0.106, −0.045)
Endorser: Mayor	−0.026***	−0.114**	−0.055***	−0.006	−0.018	−0.010
	(−0.041, −0.012)	(−0.215, −0.013)	(−0.087, −0.023)	(−0.039, 0.026)	(−0.049, 0.014)	(−0.039, 0.020)
Endorser: President	−0.037***	−0.225***	−0.071***	−0.038**	−0.045***	−0.031**
	(−0.052, −0.023)	(−0.328, −0.122)	(−0.104, −0.039)	(−0.071, −0.004)	(−0.076, −0.013)	(−0.059, −0.003)
Endorser: Right Newspaper	−0.065***	−0.267***	−0.089***	−0.063***	−0.056***	−0.060***
	(−0.080, −0.051)	(−0.369, −0.166)	(−0.121, −0.057)	(−0.096, −0.030)	(−0.087, −0.026)	(−0.089, −0.030)
Endorser: Left Newspaper	−0.060***	−0.291***	−0.096***	−0.049***	−0.049***	−0.050***
	(−0.075, −0.046)	(−0.390, −0.191)	(−0.128, −0.064)	(−0.082, −0.015)	(−0.080, −0.017)	(−0.079, −0.022)
Producer: Sinovac	−0.107***	−0.698***	−0.151***	−0.114***	−0.130***	−0.129***
	(−0.130, −0.085)	(−0.855, −0.542)	(−0.202, −0.099)	(−0.166, −0.062)	(−0.181, −0.079)	(−0.175, −0.083)
Producer: Astrazeneca	0.021*	0.182**	0.043	0.021	0.016	0.060**
	(−0.001, 0.043)	(0.037, 0.328)	(−0.009, 0.095)	(−0.030, 0.072)	(−0.034, 0.066)	(0.014, 0.106)
Producer: Pfizer	0.024**	0.265***	0.031	0.060**	0.036	0.009
	(0.002, 0.046)	(0.122, 0.409)	(−0.020, 0.082)	(0.009, 0.112)	(−0.014, 0.087)	(−0.037, 0.055)
Producer: Gamaleya	−0.054***	−0.291***	−0.027	−0.034	−0.049*	−0.035
	(−0.077, −0.032)	(−0.441, −0.141)	(−0.079, 0.024)	(−0.085, 0.018)	(−0.099, 0.001)	(−0.081, 0.011)
1% Uptake	−0.027**	−0.145*	−0.026	−0.022	−0.015	−0.004
	(−0.049, −0.005)	(−0.300, 0.010)	(−0.077, 0.026)	(−0.075, 0.031)	(−0.065, 0.036)	(−0.049, 0.041)
25% Uptake	0.002	0.147*	0.036	0.021	0.023	0.038*
	(−0.020, 0.024)	(−0.003, 0.297)	(−0.016, 0.087)	(−0.033, 0.074)	(−0.026, 0.073)	(−0.006, 0.082)
50% Uptake	0.031***	0.311***	0.084***	0.045*	0.046*	0.060**
	(0.009, 0.053)	(0.158, 0.464)	(0.032, 0.136)	(−0.008, 0.099)	(−0.003, 0.095)	(0.014, 0.105)
75% Uptake	0.053***	0.352***	0.090***	0.069**	0.053**	0.071***
	(0.031, 0.075)	(0.199, 0.506)	(0.038, 0.142)	(0.016, 0.123)	(0.003, 0.103)	(0.027, 0.115)
50% Efficacy	−0.038***	−0.301***	−0.133***	−0.128***	−0.040*	−0.051***
	(−0.056, −0.019)	(−0.440, −0.162)	(−0.175, −0.090)	(−0.171, −0.086)	(−0.082, 0.001)	(−0.088, −0.014)
70% Efficacy	−0.010	−0.094	−0.007	−0.013	0.018	−0.023
	(−0.028, 0.007)	(−0.206, 0.019)	(−0.046, 0.031)	(−0.050, 0.025)	(−0.019, 0.054)	(−0.056, 0.010)
78% Efficacy	−0.023	−0.080	−0.057	−0.046	−0.006	0.057
	(−0.073, 0.027)	(−0.429, 0.270)	(−0.168, 0.054)	(−0.135, 0.043)	(−0.099, 0.087)	(−0.036, 0.151)
91% Efficacy	0.075***	0.409***	0.090***	0.112***	0.094***	0.085***
	(0.057, 0.092)	(0.286, 0.532)	(0.053, 0.128)	(0.074, 0.151)	(0.058, 0.130)	(0.052, 0.118)
95% Efficacy	0.058***	0.319***	0.125***	0.084***	0.051***	0.083***
	(0.041, 0.076)	(0.200, 0.438)	(0.086, 0.164)	(0.044, 0.124)	(0.013, 0.089)	(0.048, 0.119)
Fixed Effects	Yes	Yes	Yes	Yes	Yes	Yes
Outcome Range	0-1	0-12	1-5	1-5	1-5	1-5
Control Mean	0.507	5.521	2.849	2.959	2.945	3.315
Control SD	0.503	4.463	1.186	1.16	1.246	1.212
Observations	31,574	31,574	31,574	31,574	31,574	31,574
R^2	0.703	0.833	0.718	0.680	0.678	0.768

Note: *p<0.1; **p<0.05; ***p<0.01

Results of the Basic Conjoint - First Round Only

The results in Table 8 show only the first-round conjoint responses, corresponding to (3). This estimator is identical to the estimator for our main analyses, except removing the individual fixed effects as we restrict our analysis to the first round from each respondent.

$$Y_{\text{irc}} = \alpha_{\text{brc}} + \beta_r Y_{\text{ic}}^{\text{pre}} + \sum_{k=1}^{4} \tau_1^k \text{Producer k}_{\text{irc}} + \sum_{k=1}^{4} \tau_3^k \text{Producer k and efficacy}_{\text{irc}}$$
$$+ \sum_{k=1}^{5} \tau_3^k \text{Endorser k}_{\text{irc}} + \sum_{k=1}^{2} \tau_4^k \text{Distributor k}_{\text{irc}} + \sum_{k=1}^{4} \tau_5^k \text{Takeup k}_{\text{irc}} + \varepsilon_{\text{irc}}, \quad (3)$$

This robustness check addresses concerns that respondents may become distracted and fail to update their responses over multiple rounds of treatments. Reassuringly, we find little difference in our point estimates as compared to the full results presented in Table 6, but as expected our estimates are less precise and therefore fewer of the estimates are statistically significant.

Supplementary Table 8: Results of the Conjoint Experiment (First Round Only)

	Dependent variable:					
	Willing	Months (Rev)	Stop Propagation	Not Get COVID	Wouldn't Harm	Gov Help
	(1)	(2)	(3)	(4)	(5)	(6)
Distributor: Civil Society	-0.034^{*}	-0.137	0.005	-0.056	-0.008	-0.029
	(0.018)	(0.120)	(0.042)	(0.041)	(0.038)	(0.041)
Distributor: Armed Forces	-0.001	-0.049	0.023	-0.030	-0.040	0.038
	(0.017)	(0.123)	(0.043)	(0.042)	(0.039)	(0.041)
Endorser: Religious Leader	-0.055^{**}	-0.312^{*}	-0.035	-0.082	-0.013	0.006
	(0.025)	(0.181)	(0.061)	(0.059)	(0.055)	(0.059)
Endorser: Mayor	-0.042^{*}	-0.394^{**}	-0.066	-0.040	-0.047	0.080
	(0.024)	(0.165)	(0.060)	(0.058)	(0.054)	(0.056)
Endorser: President	0.004	-0.201	-0.018	0.033	0.030	0.081
	(0.024)	(0.184)	(0.062)	(0.059)	(0.056)	(0.056)
Endorser: Right Newspaper	-0.068^{***}	-0.299^{*}	-0.086	-0.043	-0.027	-0.011
	(0.023)	(0.167)	(0.058)	(0.055)	(0.052)	(0.053)
Endorser: Left Newspaper	-0.049^{**}	-0.180	-0.031	-0.084	-0.037	0.048
	(0.024)	(0.167)	(0.062)	(0.059)	(0.055)	(0.058)
Producer: Sinovac	-0.123^{***}	-0.917^{***}	-0.083	-0.161^{***}	-0.166^{***}	-0.165^{***}
	(0.023)	(0.166)	(0.058)	(0.057)	(0.054)	(0.058)
Producer: Astrazeneca	0.032	0.333^{**}	0.024	0.015	0.049	0.047
	(0.024)	(0.169)	(0.062)	(0.056)	(0.056)	(0.061)
Producer: Pfizer	0.008	0.250	-0.009	0.062	0.035	-0.014
	(0.023)	(0.165)	(0.061)	(0.057)	(0.057)	(0.057)
Producer: Gamaleya	-0.048^{**}	-0.026	0.009	0.019	-0.042	0.034
	(0.023)	(0.152)	(0.059)	(0.055)	(0.053)	(0.055)
1% Uptake	0.021	0.016	0.047	0.059	0.056	-0.034
	(0.020)	(0.146)	(0.049)	(0.048)	(0.046)	(0.048)
25% Uptake	-0.006	0.086	0.023	0.028	0.019	0.019
	(0.020)	(0.136)	(0.049)	(0.047)	(0.043)	(0.047)
50% Uptake	0.005	0.281^{*}	0.061	0.065	0.024	-0.003
	(0.020)	(0.147)	(0.050)	(0.046)	(0.044)	(0.047)
75% Uptake	0.034^{*}	0.084	0.005	-0.016	-0.060	-0.008
	(0.020)	(0.144)	(0.050)	(0.049)	(0.046)	(0.046)
50% Efficacy	-0.049	-0.321	-0.149^{**}	-0.045	0.034	0.029
	(0.031)	(0.238)	(0.076)	(0.074)	(0.068)	(0.073)
70% Efficacy	-0.009	-0.314	0.099	0.026	0.067	0.035
	(0.030)	(0.209)	(0.076)	(0.071)	(0.070)	(0.075)
78% Efficacy	-0.045	-0.221	-0.175	-0.005	0.176	0.403^{***}
	(0.066)	(0.527)	(0.144)	(0.125)	(0.153)	(0.155)
91% Efficacy	0.029	-0.170	0.001	0.018	0.046	0.003
	(0.030)	(0.207)	(0.076)	(0.072)	(0.069)	(0.071)
95% Efficacy	0.043	0.087	0.077	0.003	-0.017	0.031
	(0.030)	(0.208)	(0.077)	(0.072)	(0.069)	(0.072)
Fixed Effects	Yes	Yes	Yes	Yes	Yes	Yes
Outcome Range	0-1	0-12	1-5	1-5	1-5	1-5
Control Mean	0.507	5.521	2.849	2.959	2.945	3.315
Control SD	0.503	4.463	1.186	1.16	1.246	1.212
Observations	6,489	6,489	6,489	6,489	6,489	6,489
R^2	0.350	0.598	0.171	0.167	0.165	0.200

Note: $^{*}p<0.1$; $^{**}p<0.05$; $^{***}p<0.01$

Heterogeneous Effects of Trust on Vaccine Uptake

Supplementary Table 9 plots the trust interactions which underlay Figure 3, as estimated by equation (2).

Supplementary Table 9

	Dependent variable:	
	Willing	Months (Rev)
	(1)	(2)
Civil Society × Trust	0.001	0.048
	(0.006)	(0.044)
Armed Forces × Trust	−0.007	0.030
	(0.005)	(0.039)
Religious Leader × Trust	0.015**	0.138***
	(0.007)	(0.041)
Mayor × Trust	0.020***	0.120***
	(0.006)	(0.040)
President × Trust	0.040***	0.251***
	(0.005)	(0.039)
Right-Wing Newspaper × Trust	0.018***	0.063
	(0.006)	(0.044)
Left-Wing Newspaper × Trust	0.020***	0.040
	(0.007)	(0.046)
Sinovac × Trust in China	0.047***	0.414***
	(0.007)	(0.051)
Astrazenica × Trust in UK	0.041***	0.285***
	(0.006)	(0.046)
Pfizer × Trust in Biden	0.030***	0.183***
	(0.006)	(0.044)
Pfizer× Trust in Trump	0.048***	0.307***
	(0.007)	(0.047)
Gamaleya × Trust in Russia	0.077***	0.576***
	(0.006)	(0.045)
Fixed Effects	Yes	Yes
Outcome Range	0-1	0-12
Control Mean	0.52	5.796
Control SD	0.502	4.486
Observations	31,574	31,574
R^2	0.708	0.836
Note:	*p<0.1; **p<0.05; ***p<0.01	

Effects of Political Endorsements Among Co-Partisans

Here we test the effect of political endorsements interacted with the co-partisanship of respondents. We find that co-partisans of both mayors and presidents are more positively responsive to their endorsements than non co-partisants are.

We use a special case of estimator (2) in which we interact an indicator for whether the respondent is a co-partisan of the endorser for the mayor with the mayoral endorsement, and an indicator for whether the respondent is a co-partisan of the president with the presidential endorsement. Both of these variables are drawn from pre-treatment covariates on future vote choice as reported by the respondents.

Supplementary Table 10: Effects of Political Endorsements Among Co-Partisans

	Dependent variable:	
	Willing	Months (Rev)
	(1)	(2)
Endorser: Mayor	-0.024^{***}	-0.096^{*}
	(0.008)	(0.054)
Endorser: President	-0.045^{***}	-0.282^{***}
	(0.008)	(0.055)
Vote Mayor × Mayor Endorse	0.040^{***}	0.168^{*}
	(0.013)	(0.093)
Vote President × President Endorse	0.102^{***}	0.605^{***}
	(0.015)	(0.110)
Fixed Effects	Yes	Yes
Outcome Range	0-1	0-12
Control Mean	0.507	6.109
Control SD	0.501	4.404
Observations	31,574	31,574
R^2	0.703	0.833
Note:	$^{*}p<0.1$; $^{**}p<0.05$; $^{***}p<0.01$	

Effects of Religious Endorsements Among the Religious

In one subset of our analysis, we study the effect of co-religious endorsers on vaccine uptake by co-religionists. Selecting the relevant endorser for Catholics was fairly straightforward, and we selected the Archbishop for each country in our sample. Given the more diffuse structure of evangelical churches, we consulted with local experts in religious authority within the countries in our sample. Triangulating between this and the volume of social media followers and national news coverage surrounding organizations as national-level authorities, we selected the leading national umbrella organization for evangelical groups in each country. Even so, we interpret the evangelical endorser as an in-group messenger, rather than an authority figure given nature of the church. We acknowledge that the respondent may not see the Evangelical association as their leader, but should view it as a co-religious endorser: in-group pressure versus vertical pressure.

Here we examine two subsets of religious respondents, Catholics and Evangelicals, display their sub-sample responsiveness to religious endorsers, as well as the interaction of their religious identity with religious endorsement. We find that Catholics are no more responsive to religious endorsements than the broader population. Evangelicals, on the other hand, are equally responsive to religious endorsements as to medical endorsements, and are more responsive to religious endorsements than the general population are.

We use the basic conjoint specification, equation 1 in columns (1), (2), (5), and (6), and report only the coefficient estimate for the religious leader as an endorser. In columns (3), (4), (5), and (6), we use a special case of estimator (2) in which we interact trust the religious endorser with an indicator which takes on a value of 1 if the respondent is a co-religionist of the endorser.

Supplementary Table 11: Religious Subset Analysis and Heterogeneous Effects of Co-Religious Endorsers

	Dependent variable:							
	Willing	Months (Rev)	Willing	Months (Rev)	Willing	Months (Rev)	Willing	Months (Rev)
	(1)	(2)	(3)	(4)	(5)	(6)	(7)	(8)
Endorser: Religious Leader	-0.075^{***}	-0.271^{***}	-0.071^{***}	-0.366^{***}	0.009	0.110	-0.071^{***}	-0.351^{***}
	(0.010)	(0.071)	(0.010)	(0.072)	(0.024)	(0.157)	(0.008)	(0.054)
Catholic X Co-Religious Endorser			0.005	0.069				
			(0.012)	(0.083)				
Evangelical X Co-Religious Endorser							0.045^{**}	0.350^{**}
							(0.022)	(0.142)
Sample	Catholics	Catholics	Full	Full	Evangelicals	Evangelicals	Full	Full
Full Conjoint Controls	Yes	Yes	Yes	Yes	Yes	Yes	Yes	Yes
Fixed Effects	Yes	Yes	Yes	Yes	Yes	Yes	Yes	Yes
Outcome Range	0-1	0-12	0-1	0-12	0-1	0-12	0-1	0-12
Control Mean	0.559	5.412	0.516	5.768	0.429	6.071	0.481	6.076
Control SD	0.504	4.356	0.502	4.447	0.514	5.106	0.502	4.404
Observations	17,540	17,540	31,574	31,574	3,759	3,759	31,574	31,574
R^2	0.696	0.822	0.703	0.833	0.723	0.844	0.703	0.833
Note:								*p<0.1; **p<0.05; ***p<0.01

Heterogeneous Effects - Education

Here we present heterogeneous effects of different conjoint treatment conditions by the respondents' reported education. We find that the more educated are less responsive to religious and presidential endorsements, and more responsive to higher uptake and efficacy. For these heterogeneous effects, as well as those displayed in Supplementary Table 13, we use equation 2, with education as the pre-treatment covariate.

Supplementary Table 12: Heterogeneous Effects - Education

	Dependent variable:	
	Willing	Months (Rev)
	(1)	(2)
Education x Dist.: Civil Society	−0.001	−0.020
	(0.005)	(0.036)
Education x Dist.: Armed Forces	0.002	0.029
	(0.005)	(0.038)
Education x End.: Religious Leader	−0.023***	0.004
	(0.007)	(0.053)
Education x End.: Mayor	−0.010	0.020
	(0.007)	(0.051)
Education x End.: President	−0.022***	−0.066
	(0.007)	(0.053)
Education x End.: Right Newspaper	0.0003	0.035
	(0.007)	(0.051)
Education x End.: Left Newspaper	−0.002	0.046
	(0.007)	(0.050)
Education x Prod.: Sinovac	−0.006	−0.077
	(0.010)	(0.070)
Education x Prod.: Astrazeneca	0.011	0.040
	(0.009)	(0.062)
Education x Prod.: Pfizer	0.014	−0.032
	(0.009)	(0.061)
Education x Prod.: Gamaleya	−0.009	−0.092
	(0.010)	(0.068)
Education x 1% Uptake	0.015	0.107*
	(0.009)	(0.065)
Education x 25% Uptake	0.014	0.118*
	(0.010)	(0.063)
Education x 50% Uptake	0.011	0.047
	(0.009)	(0.063)
Education x 75% Uptake	0.020**	0.185***
	(0.010)	(0.063)
Education x 50% Efficacy	−0.0003	−0.004
	(0.009)	(0.071)
Education x 70% Efficacy	0.006	−0.003
	(0.008)	(0.058)
Education x 78% Efficacy	−0.028	−0.122
	(0.026)	(0.160)
Education x 91% Efficacy	0.016*	0.067
	(0.009)	(0.065)
Education x 95% Efficacy	0.016*	0.094
	(0.009)	(0.058)
Fixed Effects	Yes	Yes
Outcome Range	0-1	0-12
Control Mean	0.507	5.521
Control SD	0.503	4.463
Observations	31,574	31,574
R^2	0.704	0.833
Note:	*p<0.1; **p<0.05; ***p<0.01	

Heterogeneous Effects - Most Hesitant Respondents

In this analysis, we define "most hesitant" as respondents who that they would wait 12 or more months prior to vaccination in the pre-treatment hesitancy questionnaire. These 'most hesitant' respondents represent 33.8% of our hesitant sample. We conduct sub-group analysis, splitting our experimental sample into the "most hesitant", as defined above, and the "less hesitant" (or respondents who would wait between 3 and 11 months to vaccinate). These results use equation 1, but subsets of the main data frame: "most hesitant" in columns (1) and (2), and "less hesitant" in columns (3) and (4).

We find that these respondents are more responsive to non-medical endorsements and lower levels of uptake, more responsive to specific than generic vaccines, and less convinced by information about higher efficacy. This table reveal that the most hesitant respondents still prefer distribution and endorsements by healthcare professionals, although this preference is less pronounced than among the less hesitant. These most hesitant respondents are indifferent across vaccines with an exception of a lower acceptance of the Sinovac vaccine, although this effect is smaller than for the less hesitant sub-population. The most hesitant are not significantly responsive to levels of community uptake. Finally, the most hesitant respond to very high levels of efficacy, but are less responsive to these efficacy levels than the less hesitant. These results indicate that endorsements by health care professionals and information about very high efficacy may somewhat reduce hesitancy among the most hesitant respondents.

Supplementary Table 13: Sub-group Analysis of Most and Less Hesitant Populations

	Dependent variable:			
	Willing	Months (Rev)	Willing	Months (Rev)
	(1)	(2)	(3)	(4)
Distributor: Civil Society	-0.012^{*}	-0.029	-0.026^{***}	-0.180^{***}
	(0.007)	(0.056)	(0.007)	(0.047)
Distributor: Armed Forces	-0.007	-0.042	-0.021^{***}	-0.056
	(0.007)	(0.057)	(0.007)	(0.048)
Endorser: Religious Leader	-0.030^{***}	-0.130^{*}	-0.088^{***}	-0.429^{***}
	(0.010)	(0.075)	(0.010)	(0.069)
Endorser: Mayor	-0.012	-0.021	-0.034^{***}	-0.160^{**}
	(0.010)	(0.077)	(0.010)	(0.066)
Endorser: President	-0.018^{*}	-0.067	-0.048^{***}	-0.310^{***}
	(0.010)	(0.081)	(0.010)	(0.067)
Endorser: Right Newspaper	-0.030^{***}	-0.103	-0.082^{***}	-0.340^{***}
	(0.010)	(0.077)	(0.010)	(0.067)
Endorser: Left Newspaper	-0.035^{***}	-0.158^{**}	-0.074^{***}	-0.354^{***}
	(0.010)	(0.076)	(0.010)	(0.065)
Producer: Sinovac	-0.044^{***}	-0.357^{***}	-0.143^{***}	-0.900^{***}
	(0.015)	(0.116)	(0.015)	(0.104)
Producer: Astrazeneca	0.025	0.053	0.015	0.226^{**}
	(0.016)	(0.119)	(0.015)	(0.093)
Producer: Pfizer	0.013	0.058	0.025^{*}	0.346^{***}
	(0.016)	(0.119)	(0.015)	(0.090)
Producer: Gamaleya	-0.002	-0.020	-0.083^{***}	-0.441^{***}
	(0.016)	(0.118)	(0.015)	(0.097)
1% Uptake	-0.016	-0.135	-0.033^{**}	-0.152
	(0.016)	(0.120)	(0.015)	(0.101)
25% Uptake	0.006	0.061	-0.001	0.186^{*}
	(0.015)	(0.115)	(0.015)	(0.098)
50% Uptake	0.020	0.137	0.035^{**}	0.392^{***}
	(0.016)	(0.121)	(0.015)	(0.099)
75% Uptake	0.020	0.110	0.069^{***}	0.463^{***}
	(0.016)	(0.118)	(0.015)	(0.100)
50% Efficacy	-0.007	-0.154^{*}	-0.051^{***}	-0.367^{***}
	(0.011)	(0.093)	(0.013)	(0.095
70% Efficacy	-0.015	-0.143	-0.007	-0.058
	(0.013)	(0.092)	(0.012)	(0.072)
78% Efficacy	-0.016	0.158	-0.027	-0.196
	(0.027)	(0.181)	(0.036)	(0.251)
91% Efficacy	0.022^{*}	0.125	0.101^{***}	0.551^{***}
	(0.012)	(0.093)	(0.012)	(0.081)
95% Efficacy	0.036^{***}	0.198^{**}	0.069^{***}	0.377^{***}
	(0.013)	(0.101)	(0.012)	(0.074)
Population	Most Hesitant	Most Hesitant	Less Hesitant	Less Hesitant
Fixed Effects	Yes	Yes	Yes	Yes
Outcome Range	0-1	0-12	0-1	0-12
Control Mean	0.172	1.483	0.727	8.182
Control SD	0.384	2.811	0.451	3.157
Observations	10,700	10,700	20,874	20,874
R^2	0.690	0.786	0.655	0.693
Note:			*p<0.1; **p<0.05; ***p<0.01	

By Country Conjoint Results

This section plots the results of our conjoint analysis subset by country. These outcome measure for these plots is willingness to vaccinate.

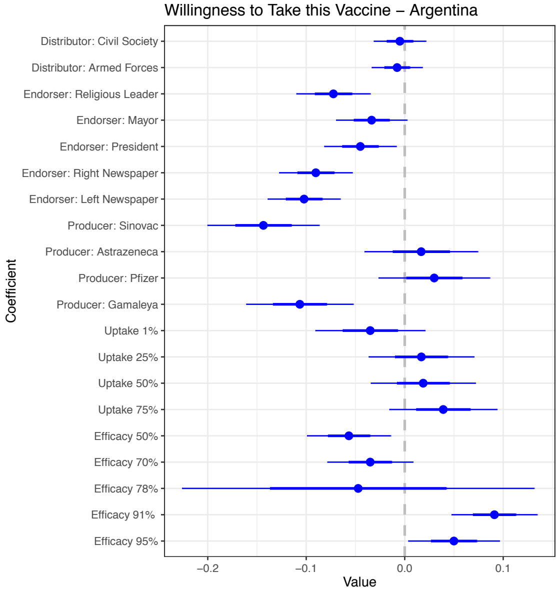

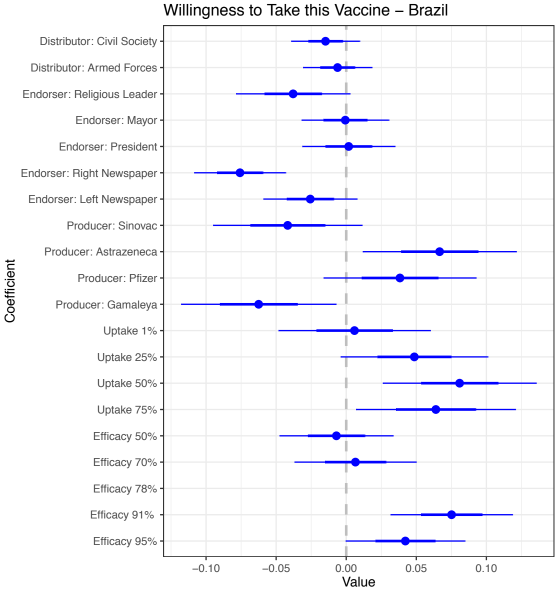

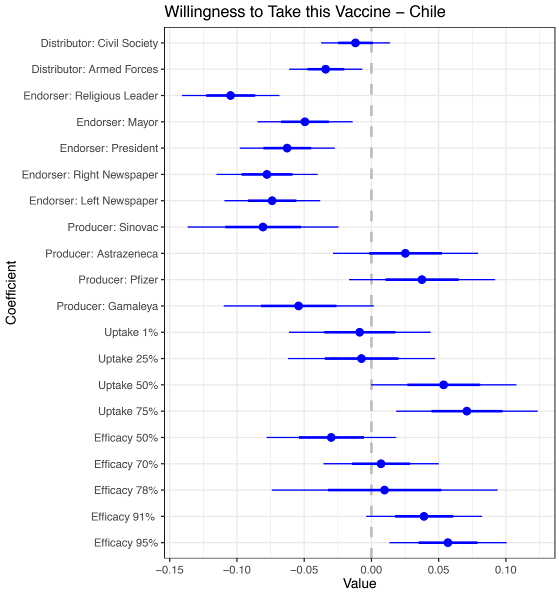

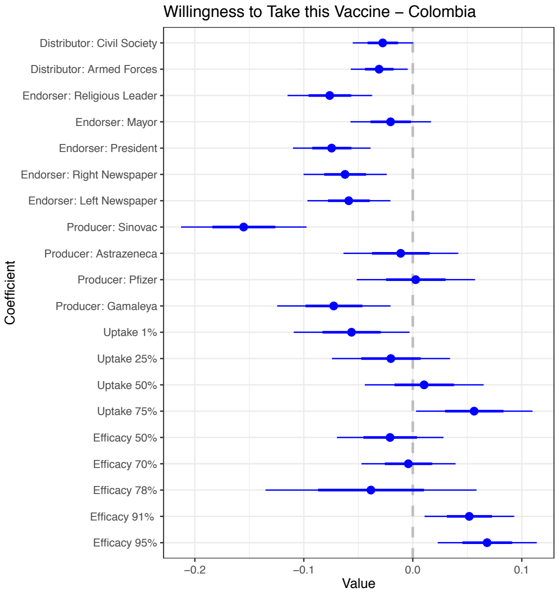

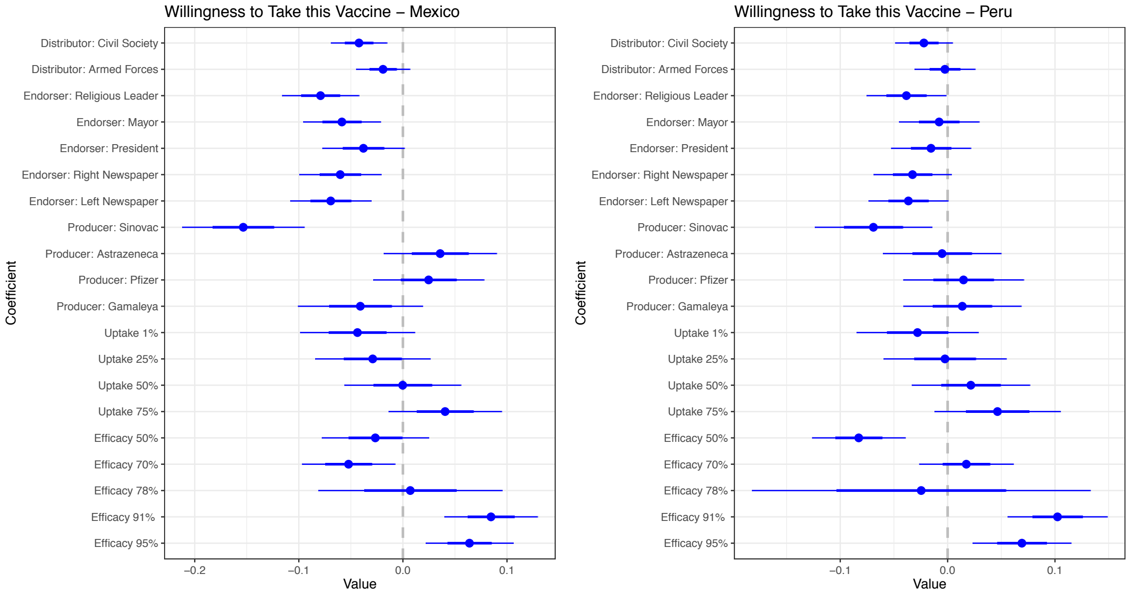

Supplementary Figure 2: By-Country Conjoint Results

By Country Trust Measures

Finally, in this section we present survey data and data from our sample exploring differences in trust across producers, distributors, and endorsers in our sample, relative to survey evidence about trust in the same in the broader population. We draw data on population-level trust from LAPOP (2020), which include nationally representative panels in each of our survey countries. Due to limitations of the LAPOP questionnaire relative to the questions we ask on trust, we only present data on questions from our survey which have an analogous question on LAPOP. We re-scale the LAPOP trust measures, which run from 1-7, to a scale of 1-4 to correspond with our questionnaire.

We find some differences cross-nationally in trust levels, as well as differences within-country comparing the vaccine hesitant population (our sample) to the broader population surveyed by LAPOP. We find that, overall, the vaccine hesitant population is less trusting of the Chinese government across all countries. We also find that in all countries except for Argentina and Peru, the vaccine hesitant are less trustful of the president of the country than the general population. Overall, cross-national variation in trust may help explain some of the variation in national level responses. Variation in trust across hesitant and non-hesitant populations may also help understand some of the pre-treatment determinants of hesitancy.

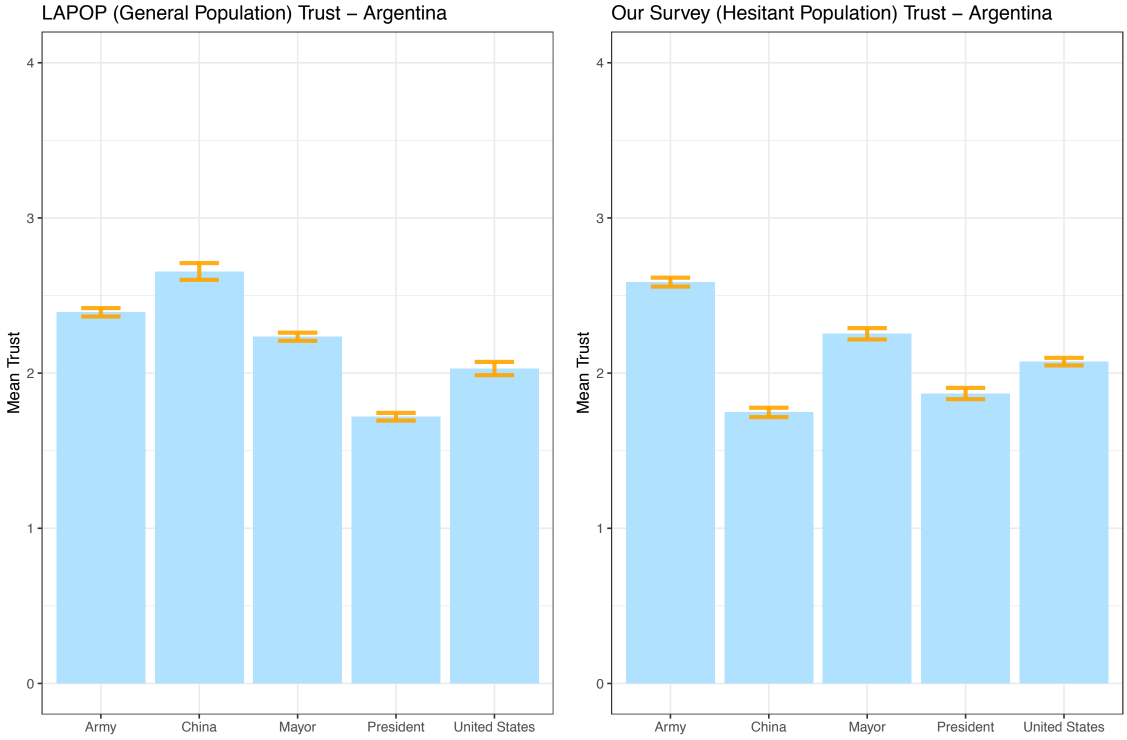

Supplementary Figure 3: Trust Measures in Argentina

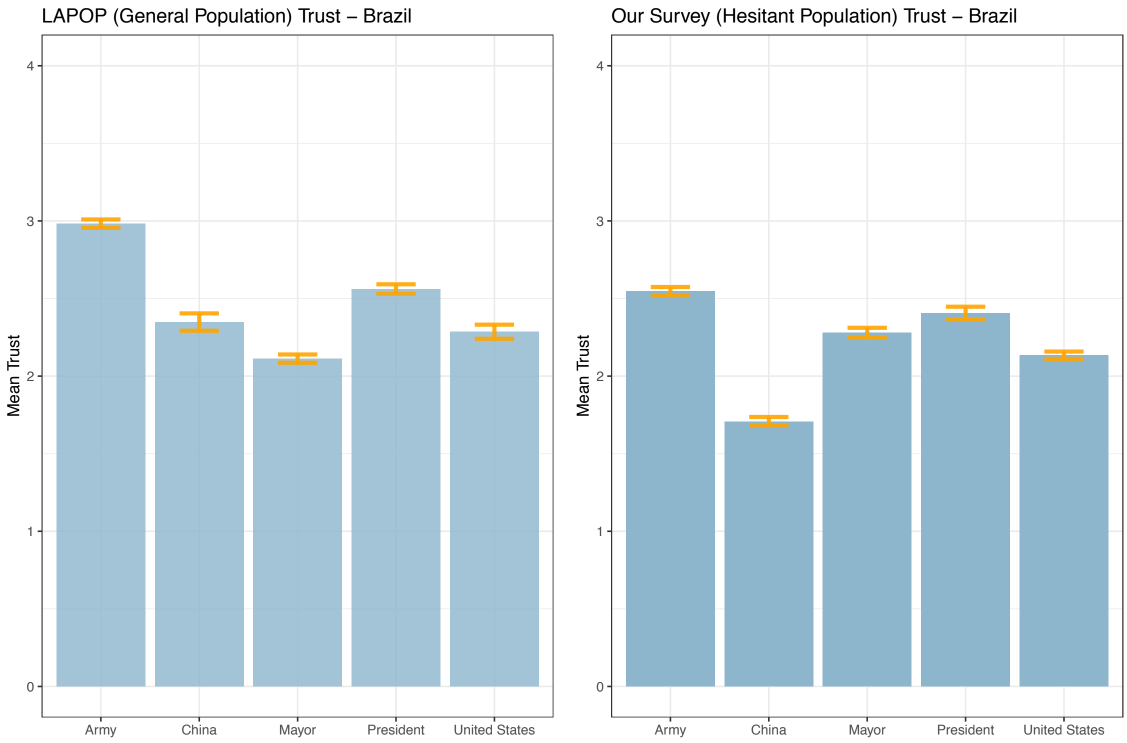

Supplementary Figure 4: Trust Measures in Brazil

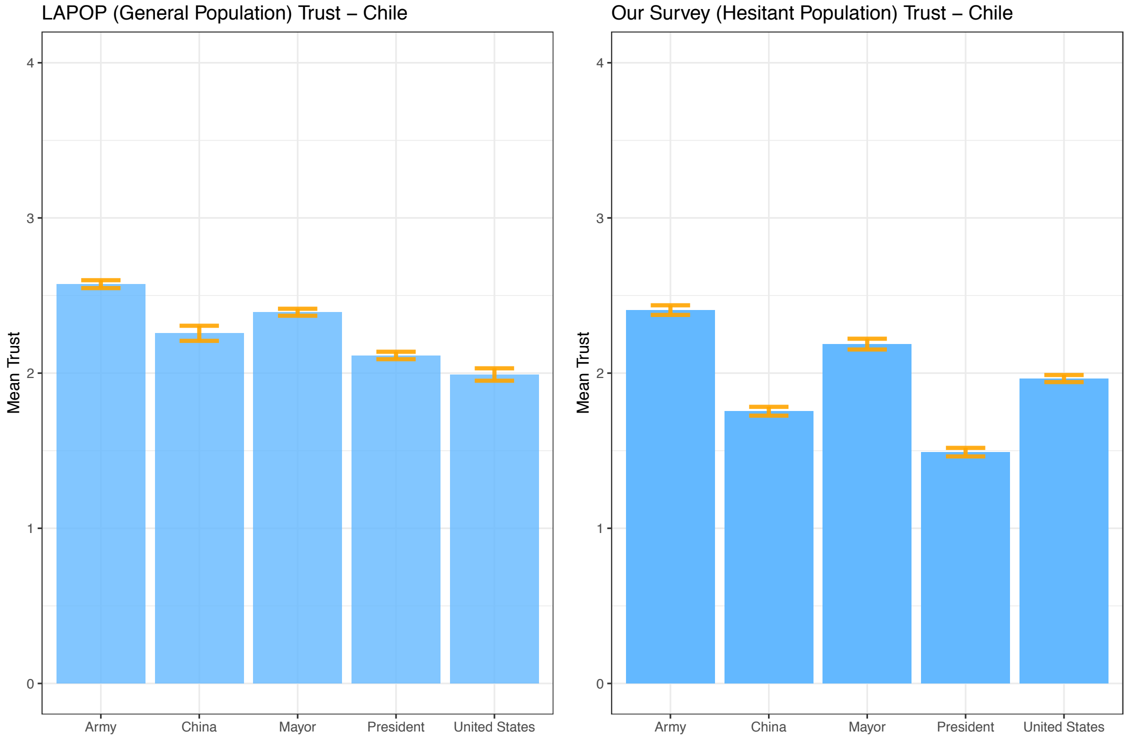

Supplementary Figure 5: Trust Measures in Chile

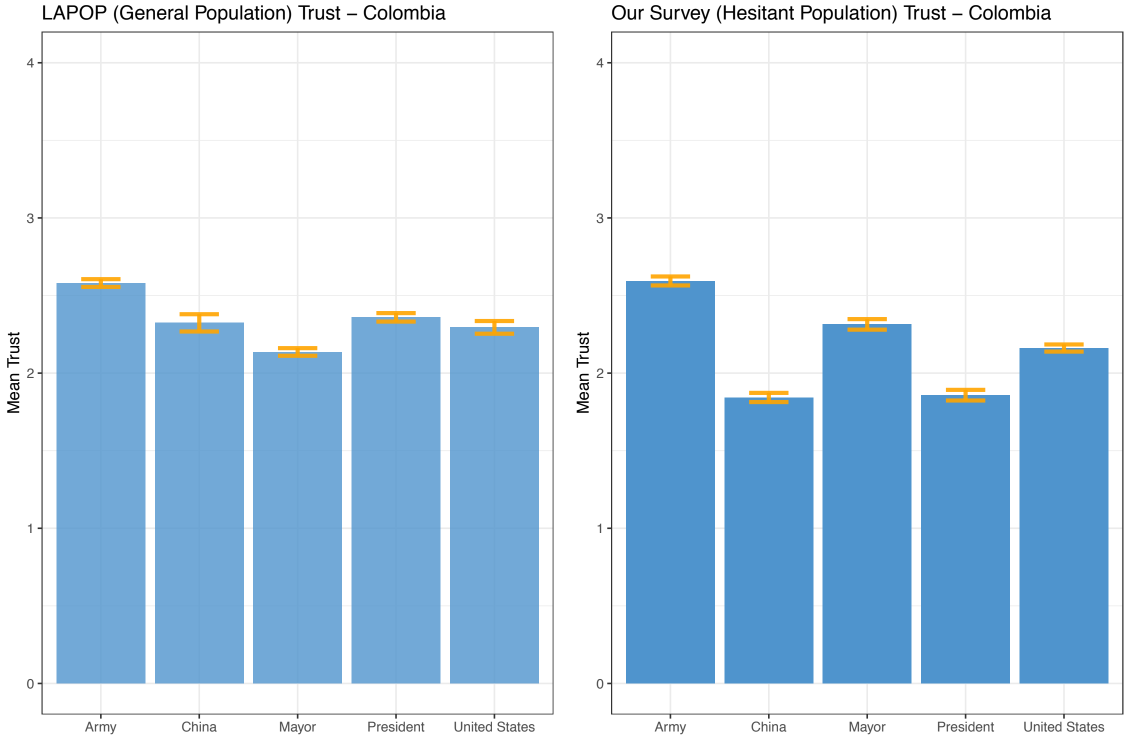

Supplementary Figure 6: Trust Measures in Colombia

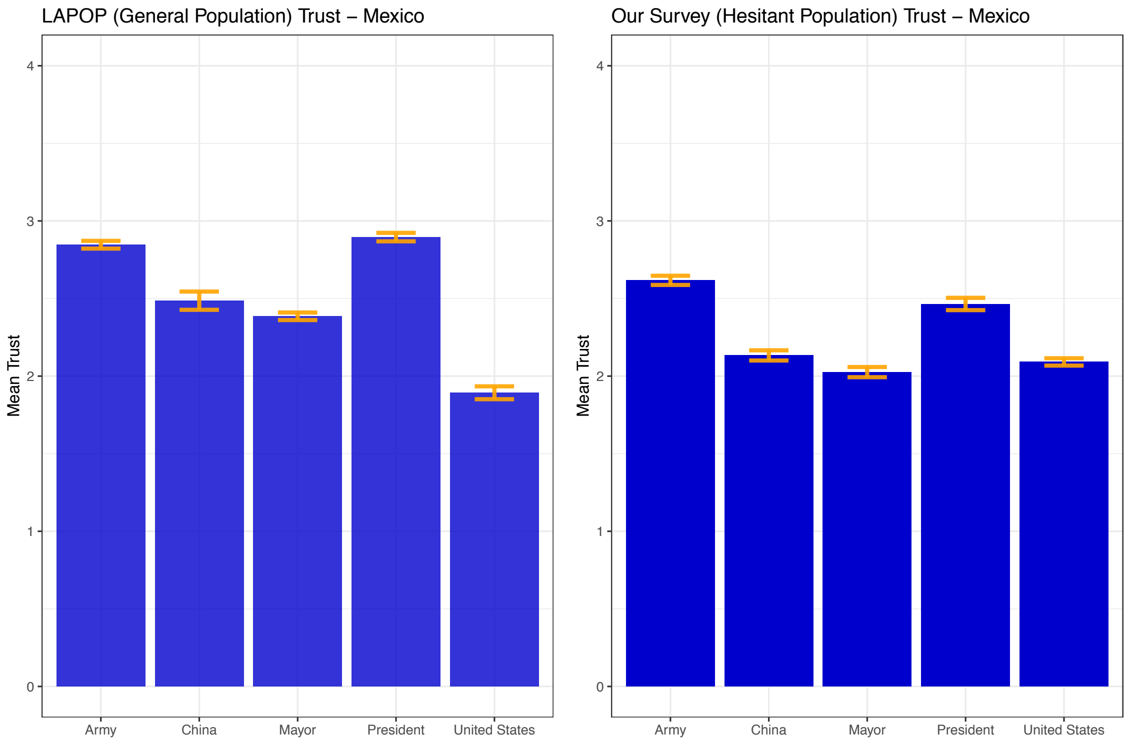

Supplementary Figure 7: Trust Measures in Mexico

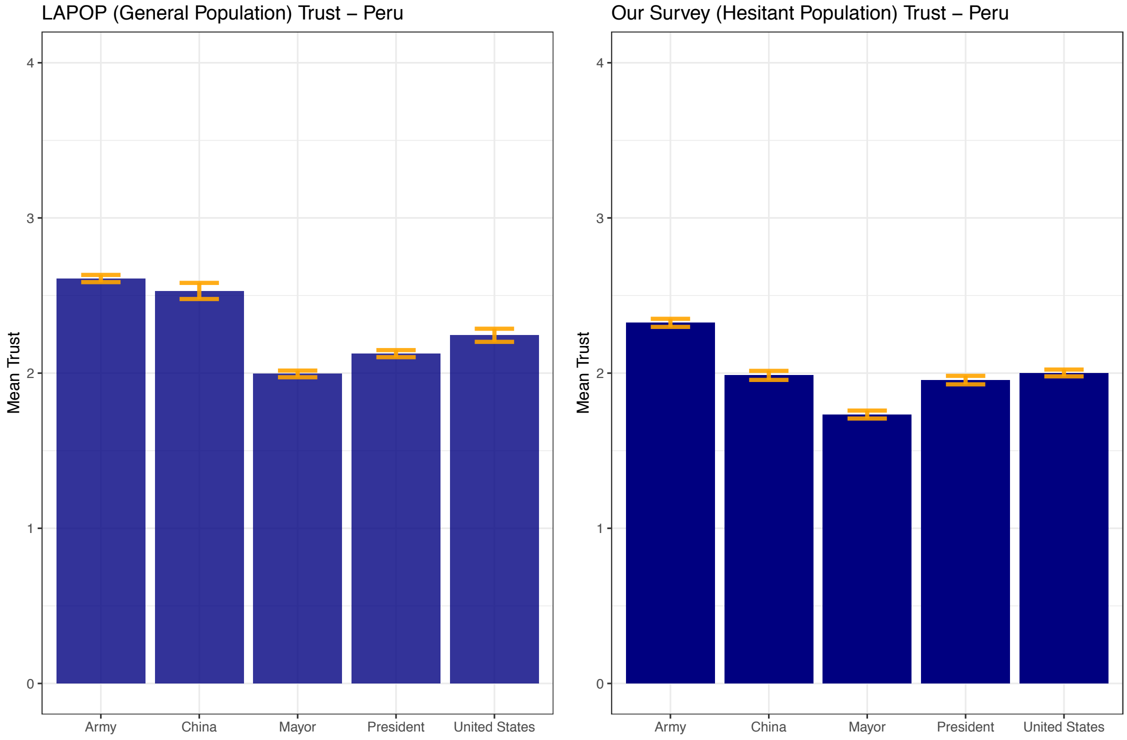

Supplementary Figure 8: Trust Measures in Peru
